# What are the experiences of people with heart failure regarding participation in physical activity? A systematic review, meta-aggregation and development of a logic model

**DOI:** 10.1136/bmjopen-2024-092457

**Published:** 2025-04-05

**Authors:** Lorna Duncan, Rosie Essery, Shoba Dawson, Yasmin Ismail, Justine Baird, Karen Butcher, Emily Whight, Rachel Johnson, Alyson L Huntley

**Affiliations:** 1 Centre for Academic Primary Care, Population Health Sciences, University of Bristol, Bristol, UK; 2 University of Southampton, Southampton, UK; 3 University Hospitals NHS Trust, Bristol, UK; 4 Sirona Care, Bristol, UK; 5 North Bristol Trust, Westbury on Trym, UK

**Keywords:** Health Equity, Systematic Review, Exercise, Heart failure

## Abstract

**Abstract:**

**Objectives:**

To (1) synthesise the experiences of people with heart failure and those who care for them concerning participation in physical activity (2) develop a logic model for a future intervention which will support people with heart failure to feel confident and safe in being physically active.

**Design:**

A systematic review and meta-aggregation using Joanna Briggs Institute (JBI) methodology.

**Data sources:**

MEDLINE, Emcare and PsycINFO databases were searched through until June 2024 inclusively.

**Eligibility criteria:**

Studies with a qualitative design, including qualitative components of mixed-methods studies, which describe experiences of participation in physical activity by adults with chronic heart failure.

**Data extraction and synthesis:**

Two independent reviewers used standardised methods to search and screen studies. Data extraction included the PROGRESS-Plus items. The JBI checklist for qualitative studies was applied. Meta-aggregation guided by JBI methods was used to synthesise the data. This evidence, along with input from a patient and public involvement group, healthcare professionals and charity organisations, was used to develop a logic model.

**Results:**

We included 28 papers (25 studies) comprising 14 qualitative studies and 11 mixed-method studies describing the perspectives of 483 people with heart failure, 64 carers and 12 healthcare professionals.

The meta-aggregation produced seven synthesised findings describing the impact of physical symptoms, emotional factors, extrinsic factors, access to knowledge, self-motivation and peer/professional motivation and the positive impact of physical activity. The PROGRESS-PLUS tool identified significant inclusivity issues within the studies. The meta-aggregation with relevant contributor input informed behavioural determinants and potential intervention components of a logic model.

**Conclusions:**

This study identifies behavioural determinants that underlie the actions of people with heart failure in their relationship with physical activity and potential intervention components for a novel intervention design to support this population. There is a lack of studies exploring health professionals’ and carers’ perspectives on this topic.

**PROSPERO registration number:**

CRD42022342883.

**Strengths and limitations of this study**

- This systematic review and meta-aggregation has followed robust methodology to produce evidence statements that can guide health practitioners and policy-makers.
- The method of using systematic review evidence to directly inform logic model content is described in detail for guidance in future similar studies.
- Despite using an equality, diversity and inclusion tool, systematic review methodology cannot overcome the poor reporting of protected characteristics in primary studies.

## Background

 For patients with symptomatic chronic heart failure (HF), physical inactivity is associated with nearly twice all-cause and cardiac mortality, and even modest exercise is associated with a survival benefit.^1^ While physical activity (PA) can improve quality of life for people with HF (PWHF), they often find it difficult to integrate self-care such as PA into their daily life.^2^ A James Lind Alliance priority setting partnership (JLA PSP) focused on advanced HF and highlighted that patients, carers and healthcare professionals (HCPs) were uncertain how PWHF should approach PA despite a willingness to do so.^3^

It is important to conduct health research according to principles of equality, diversity and inclusion (EDI).^4^ Differences such as age, gender, race and lifestyle can impact on an individual’s ability to manage their health.^5^,^8^ Historically, researchers may not have given enough consideration to the limitations of their inclusion criteria, which has restricted the profile of participants.

It is important that health research is designed to achieve maximum impact and inform healthcare practice. One way this can be achieved is by using evidence synthesis and input from relevant professional and public collaborators to develop a logic model (LM) for future intervention development.^9^ An LM is a summary of a proposed intervention’s ‘programme theory’. It describes how an intervention is expected to achieve its anticipated effects and under what circumstances.^10^ It helps us understand the issues to be addressed, determinants of relevant behaviours, potential intervention components and anticipated mechanisms to achieve the intended outcomes.^9^

## Aims

The aims were to (1) synthesise the perspectives of PWHF and those who care for them concerning participation in PA and (2) develop an LM for future intervention which will support PWHF to feel confident and safe in being physically active.

This was achieved by conducting a systematic review and meta-aggregation (MA) of relevant studies. This evidence was used to develop the LM with input from public contributors with HF, HCPs and charities to produce a framework for future intervention development.

## Methods

**Figure 1:**
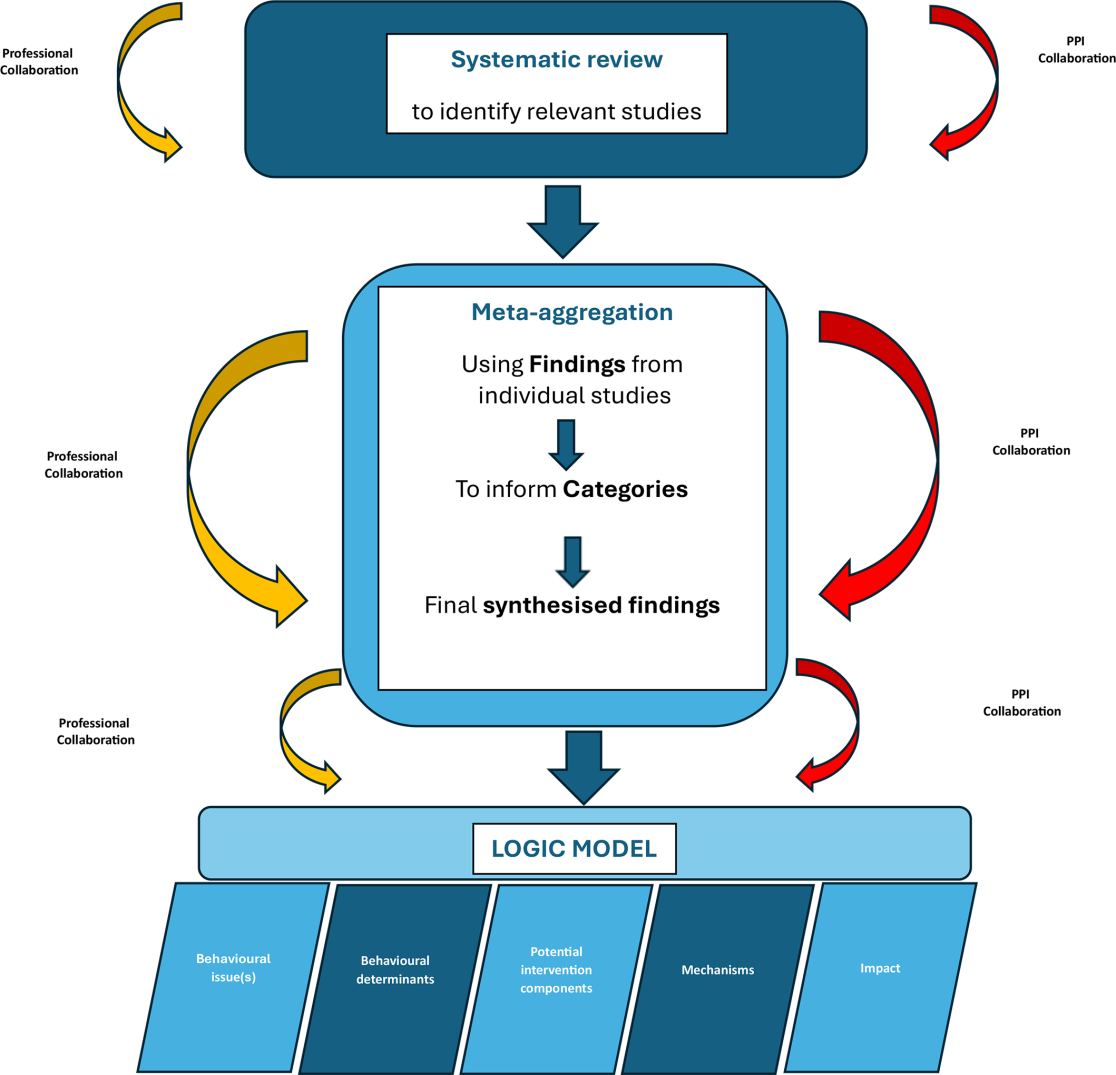
Process of systematic review, meta-aggregation and logic model development. PPI, patient and public involvement

The systematic review is reported according to Preferred Reporting Items for Systematic Reviews and Meta-Analyses guidelines^11^ and the protocol is registered on PROSPERO (unique ID: CRD42022342883).^12^ The systematic review and the evidence synthesis directly informed the LM (figure 1).

### Coinvestigators and other professional contributions

The Heart Failure and Participation in Physical activitY team comprised four researchers and five practising HF and cardiovascular HCPs from both primary and secondary care. Thus, HCP input was fully integrated into the study. During the LM development, we also contacted relevant representatives from two UK HF charities to discuss the content and any gaps in the work (see the ‘Acknowledgements’ section).

#### Public and patient involvement group contribution

A cardiologist (YI) and HF specialist nurse (EW) invited potential public and patient involvement (PPI) members. 13 PWHF agreed to contribute to the study: 3 females, 1 transgender person and 9 males covering a range of social and educational backgrounds. All were white except one contributor who was from an African-Caribbean ethnicity. Meetings throughout the study were attended by 3–5 PPI members with others contributing via email and letter. There were six PPI meetings covering: an introduction to the study, identifying any support needed, the MA, web-based charity information, the LM and a plain English summary.

#### Search strategy

The search strategy was developed and tested using MeSH and free text terms (online supplemental appendix 1). Three databases (Ovid MEDLINE (biomedicine source), APA PsycINFO (psychology source) and OVID Emcare (nursing and allied health care source) were searched from their inception to 15 July 2022. These searches were updated on 27 June 2024. This update is not reported in the Prospero methods as the protocol was registered prospectively. Rayyan software was used to screen references for inclusion.^13^

#### Eligibility criteria and screening

Studies were eligible if they considered perspectives on participation in PA by adults ≥18 years diagnosed with chronic HF of any subtype, except for those receiving palliative or end-of-life care. Using the UK Chief Medical Officer’s guidelines on PA as a reference,^14^ PA was defined as ‘any bodily movement produced by skeletal muscles that requires energy expenditure’. It was agreed that any form of PA would be included. Two reviewers (LD and SD) independently screened the titles, abstracts and full text against the criteria. Disagreements were resolved through discussion, or by inclusion of a third reviewer (AH and RJ). There were studies in which the main aim was not focused on PA. In these cases, de novo criteria were used that were not outlined in our Prospero protocol. Studies were only included if there were relevant findings or a theme based on participation in PA. Studies with isolated quotations around PA were excluded.

Reference lists and forward citations of included papers and any relevant systematic reviews were screened for potential relevance.

#### Data extraction

An Excel spreadsheet was used to extract data and encompassed the PROGRESS-plus tool.^15^ The PROGRESS-plus tool enables consideration of characteristics of study participants which stratify health opportunities and outcomes, to facilitate research inclusiveness. A Study Within A Review comparing this Cochrane PROGRESS-Plus tool and Equality Impact Assessment tool for Health Equity was registered, conducted and is reported elsewhere.^15^,^17^ Data extraction was tested and refined (LD and AH). Data were extracted by one reviewer (LD) and checked by a second (SD). Any disagreements were resolved through discussion, or by inclusion of a third reviewer (AH and RJ).

#### Data synthesis

**Figure 2:**
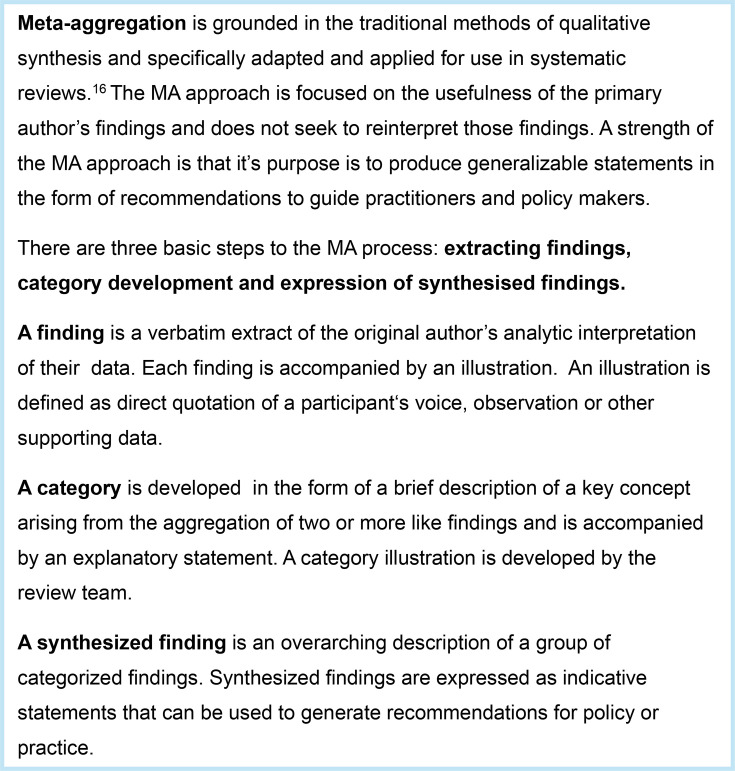
Meta-aggregation (MA) methods.

Findings related to PA were synthesised following Joanna Briggs Institute (JBI) MA methodology^18^ (figure 2).

Relevant findings/themes from each of the included studies were exported to an Excel spreadsheet (LD). The data were re-read several times across and between studies and grouped by LD into categories of related findings. The MA spreadsheet was checked, and the categories assessed for appropriateness, congruence and justification by second reviewers (SD and AH). These categories were discussed to produce the final synthesised findings in the form of statements. These synthesised findings contributed to the LM, helping to define the common determinants of PA behaviour among PWHF and also informed recommendations for potential future intervention components.

#### Critical appraisal

Critical appraisal of the included studies was undertaken by three reviewers (LD, SD and AH) using the JBI Critical Appraisal Checklist for Qualitative Research.^18^ One reviewer did the initial assessment, a second checked this, and any disagreements were resolved through discussion.

### LM development

Drawing on the results of the MA and using tools from the Person-Based Approach to intervention development,^19^ an ‘intervention planning table’ was created to document barriers and facilitators to participation in PA among PWHF. The MA evidence was mapped on to constructs of the Behaviour Change Wheel^20^ and Theoretical Domains Framework.^21^

A draft LM was created and used for a series of discussions in group and one-to-one meetings with the researchers, the HCPs coinvestigators, PPI contributors and HF charity representatives. The LM was refined on this feedback and with additional resources suggested by our contributors.^22^

## Results

### Systematic review

1108 titles and abstracts were screened against the eligibility criteria. 128 references were included for full-text screening, with 28 papers of 25 studies finally included^23^,^49^ (online supplemental appendix 2). Two intervention studies had data published across five papers.^45^,^49^

#### Characteristics of included studies

Of the 25 studies, 8 were undertaken in the USA,^3031 40 42^,^45 50^ 5 in the UK,^34 35 37 41 48 49^ 5 in Sweden,^27 28 32 38 46 47^ 3 in Australia^23 36 39^ and 1 each in Israel,^24^ France,^25^ Italy, ^26^Jordan^34^ and Norway.^29^ One study had participants from the USA and Sweden.^45^,^47^ The papers were published between 2004 and 2024.

All studies but one recruited participants with chronic HF and reduced ejection fraction. All clinical studies described participants as stable on their medication (table 1; online supplemental appendix 3). One rehabilitation study focused on participants with preserved ejection fraction, and one exercise training programme study aimed to recruit reduced ejection fraction and preserved ejection fraction participants.^36 48 49^

**Table 1:** Characteristics of studies/papers included in the systematic review

Author/yearsetting/study design	Aim	Partcipant numbers all PWHF had CHF and reduced ejection fraction except two studies where detail is given.	Agemean values unless stated	Gender/sex % female	New York HeartAssociation (NYHA) status
Qualitative interview studies (n=14)					
Albert *et al*^50^ USA SSI and CA	To learn the activity and exercise behaviours patients engaged in.	48 PWHF	<55 years=15; 55 64=17; 65=74=10; >75=6	Not reported	I=10; II=28; III=10
Amirova *et al*^23^ Australia SSI and TBF	To explore clinical, environmental, and psychosocial barriers and enablers to PA in older PWHF.	16 PWHF	79.19 (SD=5.15)	25%	I=1, II=10, III=5
Cewers *et al*^24^ Israel SSI and CI	To investigate the perceptions that HCPs may have with regard to sex differences in PA.	12 HCPs	n/a	n/a	n/a
Charuel *et al*^25^ France SSI and TA	To determine the factors that influence the practice of PA in patients with CHF managed in general practice.	19 PWHF	Range 35–94 years	53%	I=4; II=10; III=5
Durante *et al* ^26^ Italy SSI and CA	To describe caregivers contribution to HF self-care maintenance.	40 carers of 40 PWHF	Carers 53.6 (38–84 years)	20%	I=14; 11=17; III=7; IV=2
Eckerblad *et al*^27^ Sweden SSI and TA	To describe the experiences of self-care among frail, older patients with HF	19 PWHF	Median age 82 years	32%	II=4, III=11, IV=4
Europe and Tyni-Lenné^28^ Sweden SSI and CA	To get an inside perspective on how men with HF perceive their life with the illness.	20 PWHF	Range 43–73 years	0%	II=10; III=10
Markhus *et al*^29^ Norway SSI and QCA	To obtain a deeper understanding of women with HF and potential challenges relating to their sexual relationships and intimacy.	15 PWHF	Range 40–90 years	100%	II=13 III=2
Meeker *et al*^30^ USA FG and TA	To gain insight into how best to implement health coaching and public commitment strategies within the HF population.	7 PWHF(FG 1=5; FG 2=2)	57 (36–78 years)	57%	Not reported(years with HF <1 to 17.5))
Niklasson *et al*^31^ USA SSI and TA	To provide a description of how HF patients experience PA limitations in their daily lives	40 PWHF	54 (33–42) years=4; 43–52 = 13 (53–62 years) = 14 (63–73 years) =9)	73%	II=22; III=16; IV=2
Pihl *et al*^32^ Sweden SSI and PA	To describe how patients suffering from CHF conceived their physical limitations in daily life activities	15 PWHF	76 years	33%	II=3; III=11; IV=1
Saifan *et al*^33^ Jordan SSI and TA	To explore the subjective experiences of health-related quality of life among PWHF in Jordan	25 PWHF	63 (26–88 years)	48%	‘a confirmed diagnosis of HF’
Tierney *et al*^34^ England SSI and FA	To explore reasons why people with HF do and do not engage in regular PA.	22 PWHF	68.9 (53–82, SD 8.1) years	32%	I=1; II=18; III=3
Walthall *et al*^35^ UK SSI and TA	To explore the experience of fatigue and living with fatigue in persons with advanced HF	23 PWHF	72.5 53–86) years	43%	III=17; IV=6
Clinical trials with qualitative interviews (n=11)					
Adsett *et al*^36^ Australia SSI and FA	To describe motivators and barriers for people with HF participating in aquatic exercise training programmes.	14 PWHF (7 INT/7 COMP) both reduced and preserved fraction participants	70(SD 11): 74(SD 6) years	Not reported	II (6/6) ; III (1/1)
Bartlett *et al*^37^ UK SSI and realist evaluation	To evaluate the ‘Self-Management supported by Assistive, Rehabilitation and Telehealth technology’ System for CHF	7 PWHF—all INT	Not reported	Not reported	Not reported.
Hägglund *et al*^38^ Sweden SSI and CI	To evaluate Tai Chi group training among patients with CHF aged 70 years+.	10 PWHF—all INT	INT: 75.6 (71–85 years)CON 75.5 (71–83 years)	23%	75% NYHA II and III
Hwang *et al*^39^ Australia SSI and TA	To describe patient experiences and perspectives of a group-based HF telerehabilitation programme.	17 PWHF—all INT	69 years	12%	I=1; II=15; III=1
Macapagal *et al*^40^ USA SSI and PA	To explore patients’ experiences with a fitness tracker to promote ambulation before heart transplant	8 PWHF—all INT	61.75 years (SD 3.4)	13%	All=NYHA 3b-4
Okwose *et al*^41^ UK FG and TA	To identify barriers and facilitators to engagement and adherence to a home-based PA programme.	16 PWHF—all INT	67 years (SD 5.0)	19%	Not reported
Selman *et al*^42^ USA SSI and TA	To assess acceptability and appropriateness of a Tele-interventions in advanced disease populations.	12 PWHF—6 INT/6 COMP	71.2 years	75%	50% II, 50% III (INT and COMP)
Warehime *et al*^43^ USA SSI and CA	To explore factors that supported participants’ long-term exercise adherence from a larger trial focused with patients with HF.	22 PWHF—all INT	63.4 years	45%	Not reported.
Yeh *et al*^44^ USA SSI and TA	To better understand patient experiences, perceived changes, and health benefits associated with a tai chi mind-body exercise programme	32 PWHF—17 INT/15 COMP	INT 68 (SD±9) COMP (71 (±10); 66 (±7) years	41%	NYHA status:2.0 (±0.6) INT/1.7 (±0.5) COMP
Cacciata *et al*^45^ USA Klompstra *et al*^46^ Sweden Klompstra *et al*^47^ Sweden SSI and CA	To explore facilitators and challenges using a home-based exergame platform, in PWHF To describe the experiences of PWHF in INT To describe the experiences in PWHF in COMP	13 PWHF—all INT 14 PWHF—all INT 15 PWHF—all COMP	57.3±10.1 (34–69 years 70 (range 54–81 years) 60 (37–82 years)	38% 43% 40%	Mainly NYHA II All NYHA II or III II=10; III=5
Frost *et al*^48^ and Smith *et al*^49^ UK SSI and TA	To identify and explore change processes of the REACH-HF (rehabilitation) intervention. To assess the fidelity of intervention delivery and patients’ and caregivers’ experiences of participation in the REACH-HF intervention.	19 PWHF—all INT and 17 carers 15 PWHF (7 with preserved ejection fraction interviewed together with their carers	patients 68.5 years caregivers 63 years patients 70.4 years caregivers 62.8 years	37% 60%	II=13; III=6 Not reported

CAcontent analysisCHFchronic heart failureCOMPcomparator grpFAframework analysisFGfocus groupHCPshealthcare professionalsHFheart failureINTintervention grpn/anot availablePAphysical activityPAphenomenological analysisPWFHpeople with heart failureQCAqualitative content analysisSSIsemistructured questionnaireTAthematic analysisTBFtheoretical behavioural framework

The perspectives of 483 PWHF, 64 carers and 12 HCPs were extracted.

12 studies were experiential studies of PWHF and PA^2223 25 27^,^35^ and there was one experiential study each focusing solely on carers^26^ and HCPs.^24^

64 carers were included over 3 studies,^26 48 49^ although in one of these studies, the authors do not report any carers’ perspectives.^48^ The remaining 14 papers (11 studies) were intervention studies relating to PA and exercise and included the insights of PWHF.^36^,^49^

#### Characteristics of experiential studies

The 12 studies that involved PWHF focused on the broad impact of a HF diagnosis,^27^,^2933^ the symptom of fatigue,^35^ engagement in PA,^25 34 50^ the specific limitations of PA with a HF diagnosis^23 31 32^ and optimising health coaching.^30^ One study each recruited only men and only women.^28 29^ The remaining studies recruited both men and women. One study focused on caregivers’ contributions to HF self-care,^26^ and one study gathered HCPs’ perceptions on sex differences in PA.^24^

#### Characteristics of intervention studies

Interventions included aquatic and land-based training,^36^ Tai Chi^38 44^ Tele-yoga,^42^ rehabilitation,^39 41 43 48 49^ a fitness tracker,^37^ an IT tool incorporating rehabilitation, behavioural change education,^33^ exergaming^45 46^ and individual advice on PA and motivational support.^47^

#### EDI of HF participants

The Progress-plus tool gave us an indication of gaps in EDI thinking within the included papers (online supplemental appendix 3). We have not included HCP data or carer data as it was limited. Known inequities in HF research, such as a low percentage of female participants and unrepresentative age groups with biases towards a younger population were confirmed (table 1; online supplemental appendix 3).

No papers explicitly outlined EDI thinking in their methods, although age, sex and ethnic group representation are present in some of the papers’ discussion sections. In some papers, we see technological exclusion borne out by restrictive inclusion criteria for tele/remote/digital approaches (online supplemental appendix 3).

### Critical appraisal

Across the 10 questions of the JBI tool, the notable omission in required information was related to questions 6 7 and 8 in many of the studies (online supplemental appendix 4).

21/28 papers did not include a clear statement locating the researcher culturally or theoretically (Q6). 14/28 papers did not adequately report the influence of the researcher on the research, and vice-versa (Q7) and 11/28 papers did not adequately report their findings for the reviewers to be confident that all participants, and their voices were adequately represented (Q8). There were no clear differences across the qualitative interview studies and intervention studies.

### Meta-aggregation

Between 1 and 10 findings relevant to PWHF’s participation in PA were identified from each study, resulting in 115 findings (table 2).

**Table 2:** Meta-aggregation of included study findings

Finding	Category	Synthesised finding (contributing papers)
5 Findings, for example, Heart failure has a greater impact on physical activity and physical capacity than patient sex.	Physical symptoms impact on physical activity.	1. Physical symptoms impact physical activity (these may fluctuate and people with heart failure need to modify their physical activity accordingly)Experiential papers: Amirova, Cewers, Eckerblad, Europe, Markus, Meeker, Niklasson, Saifan, Tierney, Walthall.Trials with experiential data: Barlett, Selman, Warehime, Klompstra 2017, Klompstra 2021 Frost.
2 Findings, for example, Fatigue as a part of daily life	Fatigue is a specific physical barrier to physical activity.	
2 Findings, for example, Fluctuating health (symptoms; comorbid complaints; medication)	Fluctuating health and comorbidities impact physical activity.	
4 Findings, for example, Adjustment to the illness: changing lifestyle	Behaviour change is needed to manage symptoms.	
15 Findings for example, Value did not always equal motivation to move.	Motivation impacts participation in physical activity.	2. Emotional factors, impact physical activity (including low mood and fear).Experiential papers: Albert, Amirova, Cewers, Charuel, Europe, Markus, Meeker, Niklasson, Pihl, Saifan, Tierney, Walthall.Trials with experiential data: Adsett, Hagglund Hwang, Okwose, Selman, Warehime Cacciata, Klompstra 2017, Klompstra 2021 Frost, Smith.
11 Findings, for example, Not believing in one’s own ability—failing to realise their own physical capacity.	Individuality/ self-belief/loss of social role impacts participation in physical activity	
9 Findings, for example, Sense of safety.	Fear/anxiety impacts participation in physical activity	
6 Findings, for example, The surrounding environment creates barriers to increased physical activity/exercise.	Extrinsic factors impact physical activity.	3. Extrinsic factors impact physical activity for people with heart failure.Experiential papers: Albert, Amirova, Eckerblad, Meeker, Okwose, Saifan, Tierney.Trials with experiential data: Adsett, Bartlett Hwang, Selman, Klompstra 2017, Klompstra 2021 Frost.
4 Findings, for example, Life gets in the way.	Lifestyle factors impact physical activity.	
4 Findings, for example, Patients not knowing and physicians not telling.	Lack of knowledge about chronic heart failure or their condition	4. People with heart failure cannot always access knowledge(about their condition, physical activity or the benefits of regular physical activity).Experiential papers: Albert, Charuel, Markus, Meeker, Saifan.Trials with experiential data: Adsett, Hwang, Cacciata, Klompstra 2021, Frost, Smith.
1 Finding, for example, Knowing the Benefits of regular PA	Lack of knowledge about the benefits of physical activity impacts participation	
14 Findings, for example, Inclusiveness and enjoyment	Building confidence/motivation; and enablers for physical activity participation.	5. (a) Some people with heart failure are intrinsically motivated (to improve their health through physical activity). (b) For many people with heart failure, the impact of people around them, is important to their physical activity (family, friends, other people with heart failure and healthcare professionals).Experiential papers: Albert, Amirova, Durante, Eckerblad, Europe, Meeker, Saifan.Trials with experiential data: Adsett, Barlett, Hagglund, Hwang, Macapagal, Okwose, Selman, Warehime, Yeh, Cacciata, Klompstra 2021, Frost, Smith.
6 Findings, for example, Friends and family are important for the patient.	Friends and family/peers/caregivers’ impact on participation in physical activity	
11Findings, for example, Skilled and compassionate workforce.	Healthcare professionals and person-centred care impact participation in physical activity.	
7 Findings, for example, Participation prompts an increase in everyday activity levels and leads to unexpected positive outcomes.	General positive impacts of physical activity	6. Physical activity can have positive physical and psychological impacts for many people with heart failure.Experiential papers: Adsett, Amirova, Eckerblad, Markus.Trials with experiential data: Hagglund, Hwang, Macapagal, Selman, Warehime, Yeh, Cacciata, Klompstra 2021, Smith.
2 Findings: Specific to Tai Chi: for example, Perceived health-related outcomes (physical health).	Physical benefits of physical activity.	
5 Findings, for example, Experienced psychosocial benefits on stress, mood and family interactions.	Psychological and social benefits of physical activity	
7 Findings, for example, Suggestions for improvements and advice for others.	Challenges/null effects of participating in physical activity	

The experiential studies had a greater focus on activities of daily living and self-directed PA, while the intervention studies were based around structured or directed exercise. When the study findings were grouped into common categories both types of study were represented.

When synthesised, 18 categories were identified (table 2). These categories were discussed by the team to produce seven synthesised findings expressed as statements, with indicative participant quotations. The sex and age of participants are included where available to contextualise the quotations.

#### Physical symptoms impact PA (these may fluctuate, and PWHF need to modify their PA accordingly)

16 papers (15 studies) contributed to this synthesised finding.^2324 27^,^31 33^ It covers present and past experiences of PA both around exercise and general everyday activity for PWHF.

The synthesised finding describes the need for adjustment around being physically active following diagnosis. This is challenged by fluctuating health, with fatigue being an important limiting symptom, as is the influence of comorbidities.

I’ve got muscle pains in my legs, which is a nuisance, well more than a nuisance …. I am as active as I can be you know … .for some reason I can get on a bike and cycle, but I can’t walk

‘Male in early 70s’ (Walthall)

I try to grocery shop …. there are so many times when I actually have to go sit down, because I can’t make it all the way through the shop.

‘Participant in late 40s’ (Niklasson)

The side effects of medication can also impact on the PA of PWHF.

I don’t [go for walks] because this water retention tablets make me very sick, made my stomach very upset. [] Two, three steps…. I am afraid I will fall because of this medication.

No participant details (Amirova)

#### Emotional factors impact PA (including low mood and fear)

23 papers (20 studies) contributed to this synthesised finding.^23^,^2528^ It describes emotional barriers to PA which are closely linked to physical barriers in the first synthesised finding.

This female participant’s quotation below describes the cessation of her sex life following diagnosis. The importance of this topic was also highlighted by the PPI contributors and one of the heart specialist nurse contributors. Such a change in a relationship is likely to have an emotional impact on both PWHF and their partner/spouse.

All sexual activity ceased on the day the diagnosis was confirmed, because he was afraid of worsening my HF condition

‘Female in early 70s’ (Markus)^29^

The experience of exacerbating symptoms or the fear of exacerbating symptoms is cited as a barrier by some participants. This was closely linked to the lack of certainty about how to safely engage in PA despite the desire by participants to improve their health. This makes it hard for PWHF to believe in their ability to be physically active.

Every time I exercised, I felt horrible. I thought I was going to die, or have a heart attack, or something.

No participant details (Meeker 2019)

I can’t walk a lot, you see, I can’t go far. I don’t know. Am I scared? I think that, without realizing it, yes. I have a neighbor not far away that I like, well I am a little scared of going to their home.

‘Female aged 75–94’ (Charuel 2022)

As for physical barriers, if PWHF can be supported and face their emotional barriers, we see the benefits of PA described in the seventh synthesised finding. In some cases, emotional states can provide motivation, such as enjoyment of specific activities. This is described best in the fifth synthesised finding below.

#### Extrinsic factors impact PA for PWHF

14 papers (13 studies) contributed to this synthesised finding.^2327 30 33 34 36 37 39 40 42 46^,^48 50^ It includes factors as broad as living conditions, the weather and costs of PA.

The participant quoted below could not physically cope with living in their home but also describes the emotional impact of having to move. Thus, this impact is also relevant to the first and second synthesised findings.

I used to live in the third floor with no elevator. I could not go upstairs. I moved out to a first floor in another house. This was overwhelming.

‘Participant 3’ (Saifan)

Participation in PA can be impacted by the weather and also by an individual’s physical response to the environment. The second quotation below overlaps with the first synthesised finding in that the participants’ comorbidities are impeding their PA.

I think some of the benefit was the climate as well, it’s a struggle in wind and rain.

‘Female, early 60s’ (Okwose)

Because of my allergies and asthma, this time of the year from March until maybe July I have to walk indoors at the mall

‘Female, 70–75 years’ (Selman)

The cost of being physically active is an important factor and potential barrier to many. It is possible that a diagnosis of HF could impact on income, although this did not come across strongly in the synthesis as the majority of the participants were retirement age and above. The importance of cost was reinforced by our PPI contributors.

I used to go swimming and things like this… now they won’t allow any concessions for people to go swimming and things like that. I mean, it’s about 3 or 4 pounds… for about a 20 minute swim, so I won’t go….

‘Participant 3’ (Tierney)

#### PWHF cannot always access knowledge (about their condition, PA or the benefits of regular PA)

11 papers (9 studies) contributed to this synthesised finding.^2325 30 33 36 39 45 47^,^50^ It reinforces the importance of the role of HCPs in supporting PWHFs’ participation in PA. This lack of certainty about PA, and the perceived lack of advice about appropriate frequency, intensity, duration and type of PA is a key barrier for PWHFs engaging in, and sustaining PA. This links with some of the emotional impacts around safety described in the second synthesised finding.

… if you don’t have that guidance, it’s really hard because the doctors sent me out of the hospital and said, ‘Okay, now you’ve got to exercise. You’ve got to eat right.

No participant detail (Meeker)

Patients could feel insecure, as they expressed not knowing how much physical activity they could perform, or which kinds of physical activity they could perform.

Authors’ finding (Klompstra)

These experiences emphasise the importance of relaying practical, safe information around participation in PA and its benefits, as well as HCPs being able to answer questions and reassure their patients. The PPI contributor discussions reinforced the importance of this support.

#### Some PWHF are intrinsically motivated (to improve their health through PA)

#### For many PWHF, the impact of people around them, is important to their PA (family, friends, other PWHF and HCPs)

20 papers (18 studies) contributed to these inter-related synthesised findings.^2326^,^28 30 33 36^ The importance of enjoyment and sense of achievement is an important motivator of PA behaviour.

I started with 695 steps… but I’m up to—my highest I think is 5000 steps, but I’m going to beat that today. This is a motivator; you really want to take one more step than you did yesterday. If you are a competitive person like me

No participant detail (Macapagal)

For some people, this is not possible without a support network. This can be driven by HCPs or by peer or family support. A spouse or family support was an important factor raised by our PPI group and was cited when the research team asked the group about EDI considerations.^17^

Definitely the support group, ’cause they’re the peers who really get the struggle and they’re living it, and they can relate. ’Cause friends who haven’t had congestive heart failure don’t understand.

No participant detail (Meeker)

I’m trying to be as physically active as I possibly can. Me and my wife do Nordic walking almost every day.

‘Male, early 80s’ (Eckerblad 2023)

#### PA can have positive physical and psychological impacts for many PWHF

13 papers (12 studies) contributed to this synthesised finding.^2327 29 36 38^,^40 42^ Participation in PA appears to prompt an increase in everyday activity levels with many positive benefits including psychosocial outcomes. There is overlap with the sixth synthesised finding around peer support.

I don’t have anxiety very much anymore, I mean, I get bouts of it now and then but overall, it’s helped me.

No participant detail (Warehime)

I liked the program because I felt my health has improved. Before, let me tell you something, before I used to do 3 laps around the house, and I would have to stop. Now I can do 10 laps and I don’t feel tired

No participant detail (Hwang)

There was limited or no evidence for any negative impacts of supported PA for PWHF. However, it is important to consider the barriers cited in the first and second synthesised findings, and initial engagement in PA.

### Logic model

**Figure 3:**
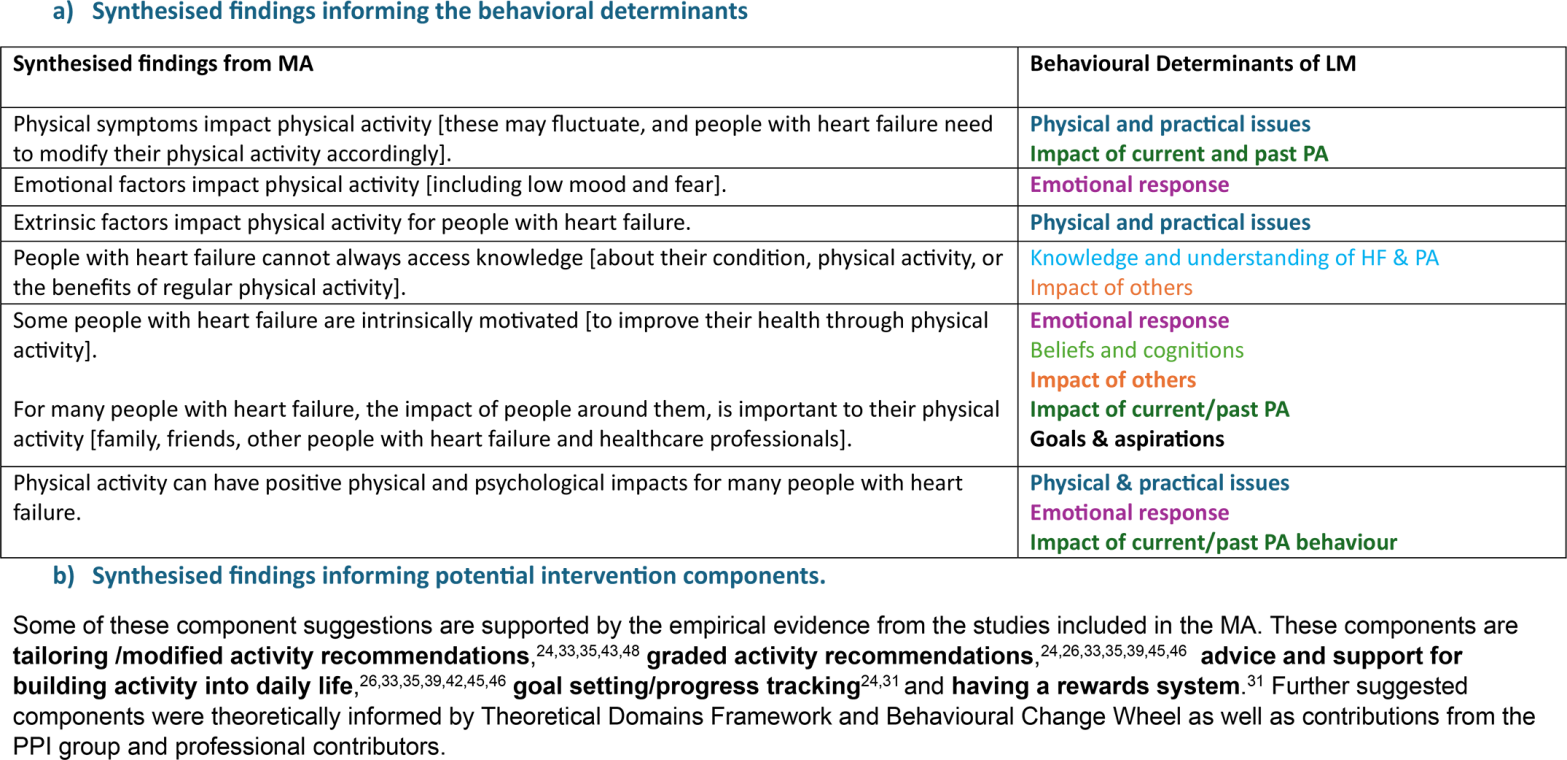
Development of the logic model (LM). HF, heart failure; MA, meta-aggregation; PA, physical activity.

The synthesised findings from the MA informed our initial framework for the behavioural determinants of the LM (figure 3). Combining the MA findings with the theoretical mapping process also provides potential ways to address some of the barriers and facilitators to behaviour by proposing potential intervention components (figure 3).

**Figure 4:**
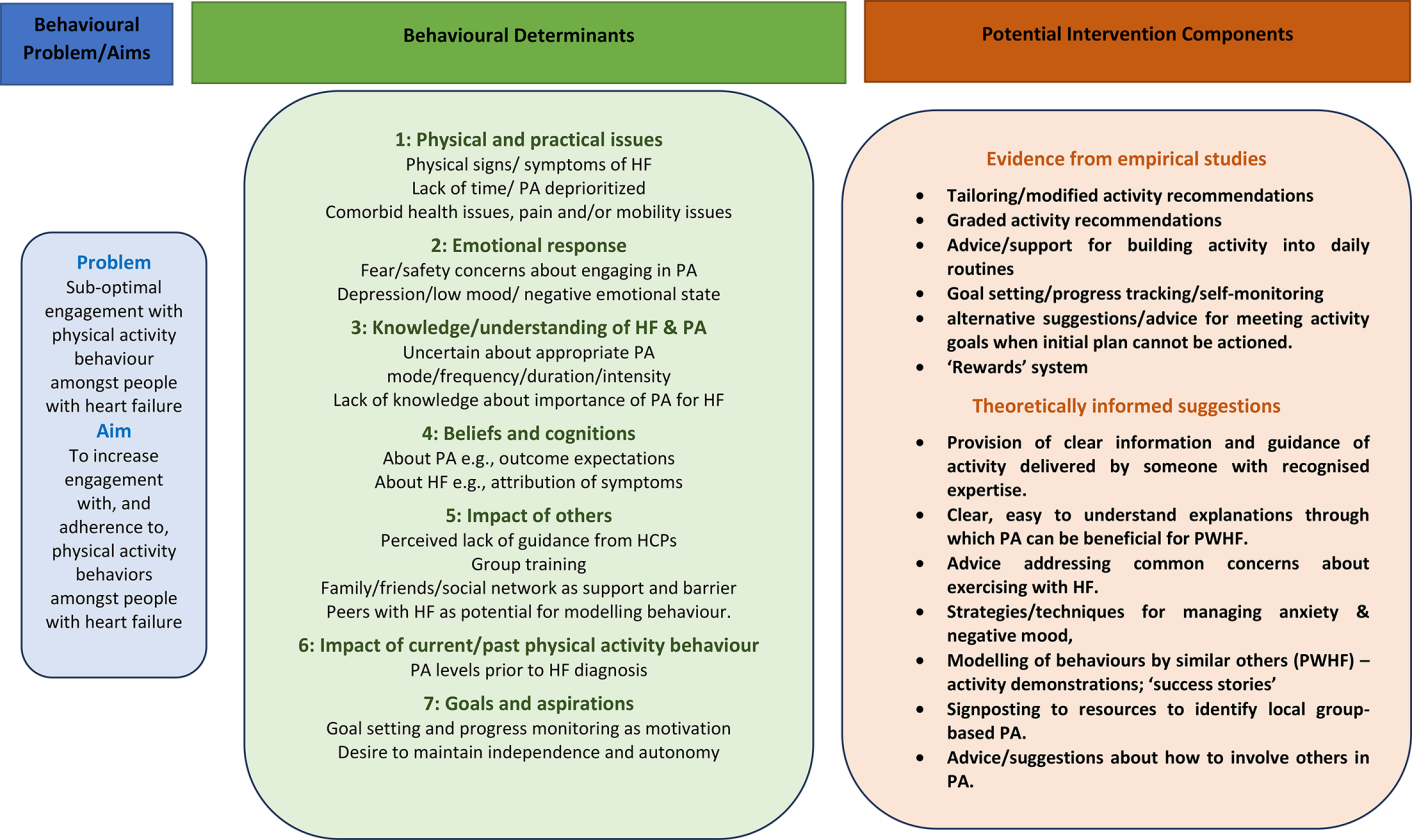
Logic model. HCPs, healthcare professionals; HF, heart failure; PA, physical activity; PWHF, people with heart failure.

The final LM outlines: (a) the behavioural issue to be addressed that is, suboptimal engagement in PA behaviour among PWHF; (b) the evidence-based determinants of this behaviour and (c) theory and/or evidence-based potential intervention components that may address these determinants (figure 4).

## Discussion

This systematic review and MA has identified physical, emotional and practical factors for PWHF around their participation in PA. The MA along with relevant contributor input has facilitated the construction of an LM for a potential future intervention to support PWHF in this regard.

This study was motivated by our previous JLA PSP which strongly indicated that many PWHF are keen to be physically active, but want and need the support of HCPs for guidance and reassurance.^3^ This is also supported by research around self-management and self-efficacy of PWHF.^2^ In the same PSP, HCPs expressed the challenges of providing this support. A previous review on HCP interaction with PWHF supports the need for this approach.^51^

Previous research has focused on PA intervention efficacy and functional outcomes.^52^ This MA shows that the complementary qualitative research is embedded within the broader literature and within specific trial settings.^23^,^50^

A systematic review by Tierney *et al* investigated exercise adherence factors for PWHF, concluding long-term commitment was challenging.^53^ Platz *et al* examined the qualitative evidence around alternative rehabilitation interventions delivered to cardiac patients including PWHF, to determine barriers, facilitators and benefits.^54^ A further 2023 Bayesian meta-analysis identified behavioural change components for intervention design to support PA in PWHF.^55^

These data concur with and support our research, which adds to this by focusing on supporting PWHF in everyday PA via HCPs and their social network. It is also inclusive of PWHF whether they have or have not accessed or engaged with cardiovascular rehabilitation.

Access to rehabilitation for PWHF across different healthcare settings in the UK and internationally varies,^56^ and uptake and completion are consistently low in the UK.^57^ In the UK and other settings, there are many PWHF who are cared for in the community and are likely to be older and with multiple long-term conditions, for whom there is no clear pathway to support their concerns around PA.

This study identifies important evidence gaps. First, it reinforces the known issues around inclusion criteria of PWHF in research, namely unrepresentative profiles around age, gender, and in some cases, ethnic and cultural diversity. We recommend that cardiovascular evidence synthesis research needs to report equality issues using the Progress-plus tool or its update PRO-EDIT to keep these inequalities on the research agenda.^58^

Second, there is a lack of studies on the role of community HCPs and an individual’s support network to influence their ability to be active.

Third, this work highlights the exclusion of people with cognitive impairment and those lacking technological/digital access and abilities. This brings up the broader topic of technology for supporting PWHF in their participation in PA. Regardless of the age or other characteristics of PWHF, if they have cognitive impairment, no access to technology or no desire to engage with it, this not only potentially excludes them from research studies but also from real-world provision.

The standalone qualitative studies in the review did not address this issue, but some of the clinical studies did for example, telerehabilitation, apps, fitness trackers.^3739 40 42 45^,^47^ These suggest that overall, these interventions were acceptable, but there were also barriers expressed, for example, difficulty in setting up, ‘boring’ and less social support. Technological/digital facilitation or exclusion is an important consideration in the care of any population including PWHF and PA and needs to be addressed.

The systematic review, MA and LM development were conducted using established robust methodological approaches. The methods were strengthened by the perspectives of PPI, HCPs and charities. We considered EDI principles and achieved diversity within the PPI group.

It is important to note that the most commonly cited NYHA (New York Heart Association) status for participants in the studies was II and III, and only one study focused on PWHF with preserved ejection fraction. This must be taken into account when considering the conclusions. The publication date of some studies meant they would not have considered including EDI issues within their reporting.

This study proposes that support, guidance and reassurance for PWHF around PA and their support network should be accessible and embedded into the ongoing care of this population, whether via primary/community care or outpatient care. This approach has the potential to impact the quality of life of PWHF and to reduce healthcare burden.

In conclusion, this study used systematic review, MA and LM methodology to identify behavioural determinants that underlie the actions of PWHF in their relationship with PA and specified potential intervention components of a service to support PA for PWHF. Inclusivity and fair representation for PWHF within research remain a concern.

## supplementary material

10.1136/bmjopen-2024-092457 online supplemental file 1

10.1136/bmjopen-2024-092457 online supplemental file 2

10.1136/bmjopen-2024-092457 online supplemental file 3

10.1136/bmjopen-2024-092457 online supplemental file 4

## Data Availability

Data are available on reasonable request. All data relevant to the study are included in the article or uploaded as supplementary information.
