# Supplementary material for: What are the experiences of people with heart failure regarding participation in physical activity? A systematic review, meta-aggregation and development of a logic model
Source: BMJ Open. 2025 Apr 5;15(4):e092457. doi: 10.1136/bmjopen-2024-092457 (PMC11973767; doi:10.1136/bmjopen-2024-092457)
Supplement: online supplemental file 1 [file bmjopen-15-4-s001.pdf]

Appendix 1 - Search strategies for Ovid MEDLINE, APA PsycInfo and Ovid Emcare databases

Database: Ovid MEDLINE(R) <1946 to present> Search Strategy:

1 exp heart failure/
2 (heart adj2 failure*).tw.
3 diastolic dysfunction.tw.
4 systolic dysfunction.tw.
5 congestive heart.tw.
6 LV dysfunction.tw.
7 left ventricular dysfunction.tw.
8 left ventricular impairment.tw.
9 systolic impairment.tw.
10 diastolic impairment.tw.
11 (Heart failure with preserved ejection fraction or HFPEF).tw.
12 (Heart failure with reduced ejection fraction or HFREF).tw.
13 or/1-12
14 Exercise/ or Exercise Therapy/ or Exercise Movement Techniques/ or Exercise Tolerance/ or Resistance Training/
15 Rehabilitation/ or Physical Therapy Modalities/
16 "Activities of Daily Living"/
17 (physical activit* or motor activit* or outdoor activit* or movement or sexual activit* or exercis* or active transport* or active living or leisure activit* or fitness or acceleromet* or walk* or lifestyle* or behavio* change* or behavio* intervent* or sport*).mp.
18 (movement or gaming or fitbit or pedometer).mp.
19 Self-Care/ or Self-Management/ or Motivation/
20 (self-care or self-management or motivation).tw.
21 Mind-Body Therapies/ or Tai Ji/ or Yoga/ or Muscle Stretching Exercises/
22 Sexual Behavior/
23 (well-being or social prescribing).tw.
24 (belief* or perception* or attitude* or perspective*).tw.
25 or/14-24
26 Qualitative Research/
27 Focus groups/
28 (ethnographic research or ethnological research or observational method* or semi-structured questionnaire* or purposive sampl* or phenomenolog* or mixed methods).mp.
29 (patient narrative or patient experience).mp.
30 (discourse analysis or content analysis or constant comparative method or Nvivo).mp.
31 or/26-30
32 13 and 25 and 31
33 limit 32 to humans

Database: APA PsycInfo <1806 to July Week 2 2022> Search Strategy:
1 (heart adj2 failure*).tw.
2 diastolic dysfunction.tw.
3 systolic dysfunction.tw.
4 congestive heart.tw.
5 LV dysfunction.tw.
6 left ventricular dysfunction.tw.
7 left ventricular impairment.tw.
8 systolic impairment.tw.
9 diastolic impairment.tw.
10 or/1-
11 Exercise/ or Activity Level/ or Physical Activity/
12 Physical Therapy/ or Physical Mobility/ or "Activities of Daily Living"/ or Rehabilitation/
13 (physical activit* or motor activit* or outdoor activit* or exercis* or active transport* or movement or sexual activity or active living or leisure activit* or fitness or acceleromet* or walk* or lifestyle* or behavio* change* or behavio* intervent* or sport* or resistance training).mp. [mp=title, abstract, heading word, table of contents, key concepts, original title, tests & measures, mesh word]
14 (gaming or fitbit or pedometer).mp.
15 (mind-body therap* or tai chi or tai ji or well-being).mp. [mp=title, abstract, heading word, table of contents, key concepts, original title, tests & measures, mesh word]
16 Self-Care/ or Self-Management/ or Motivation/
17 (self-care or self-management or motivation).tw.
18 Mind Body Therapy/ or Yoga/
19 (mind-body therap* or tai chi or tai ji or well-being or social prescribing).mp.
20 Animal Sexual Behavior/
21 (belief* or perception* or attitude* or perspective*).tw.
22 or/11-21
23 Qualitative Methods/
24 Focus Group/
25 Ethnography/
26 Observation Methods/
27 Phenomenology/
28 Narratives/
29 (ethnographic research or ethnological research or observational method* or semi-structured questionnaire* or purposive sampl* or phenomenolog* or mixed methods).mp.
30 (patient narrative or patient experience).mp.
31 Thematic Analysis/
32 Discourse Analysis/
33 Content Analysis/
34 (constant comparative method* or Nvivo).mp. [mp=title, abstract, heading word, table of contents, key concepts, original title, tests & measures, mesh word]
35 or/23-34
36 10 and 22 and 35
37 limit 36 to human

Database: Ovid Emcare <1995 to 2022 Week 27> Search Strategy: **15-07-22**
1 exp heart failure/
2 (heart adj2 failure*).tw.

3 diastolic dysfunction.tw.
4 systolic dysfunction.tw.
5 congestive heart.tw.
6 LV dysfunction.tw.
7 left ventricular dysfunction.tw.
8 left ventricular impairment.tw.
9 systolic impairment.tw.
10 diastolic impairment.tw.
11 (Heart failure with preserved ejection fraction or HFPEF).tw.
12 (Heart failure with reduced ejection fraction or HFREF).tw.
13 or/1-12
14 exercise/ or "physical activity, capacity and performance"/ or physical activity/
15 rehabilitation/
16 daily life activity/
17 (physical activit* or motor activit* or outdoor activit* or movement or sexual activit* or exercis* or active transport* or active living or leisure activit* or fitness or acceleromet* or walk* or lifestyle* or behavio* change* or behavio* intervent* or sport*).mp.
18 (gaming or fitbit or pedometer).mp.
19 self care/ or motivation/ or intrinsic motivation/ or extrinsic motivation/
20 (self-care or self-management or motivation).mp.
21 Tai Chi/ or Yoga/ or muscle stretching/ or stretching exercise/
22 (tai chi or tai ji).mp.
23 sexual behavior/
24 (well-being or social prescribing).tw.
25 (belief* or perception* or attitude* or perspective*).tw.
26 or/14-25
27 qualitative research/
28 ethnography/ or ethnology/ or phenomenology/ or narrative/ or observational method/
29 (focus group* or purposive sample or semi-structured questionnaire or mixed methods).mp.
30 (patient narrative or patient experience).mp.
31 qualitative analysis/ or discourse analysis.mp. or thematic analysis/ or content analysis/ [mp=title, abstract, heading word, drug trade name, original title, device manufacturer, drug manufacturer, device trade name, keyword heading word]
32 (discourse analysis or content analysis or constant comparative method or Nvivo).mp.
33 or/27-32
34 13 and 26 and 33
35 limit 34 to human
