# Supplementary material for: What are the experiences of people with heart failure regarding participation in physical activity? A systematic review, meta-aggregation and development of a logic model
Source: BMJ Open. 2025 Apr 5;15(4):e092457. doi: 10.1136/bmjopen-2024-092457 (PMC11973767; doi:10.1136/bmjopen-2024-092457)
Supplement: online supplemental file 2 [file bmjopen-15-4-s002.docx]

**Appendix 1. PRISMA flow diagram**

**Identification of studies via databases and registers**

Records removed *before screening*:

Duplicate records removed

(n = 435)

Records marked as ineligible by automation tools (n = 0)

Records removed for other reasons (n = 0)

Records identified from:

Databases:

Medline (n = 607)

Emcare (n = 818)

PsycInfo (n = 118)

**Identification**

Records screened.

(n = 1,108)

Records excluded.

(n = 980

including 39 systematic reviews)

Reports sought for retrieval.

(n = 128)

Reports not retrieved.

(n = 0)

**Screening**

Reports excluded:

(n=100)

Reports assessed for eligibility.

(n = 128)

Studies included in review.

(n = 25)

Reports of included studies.

(n = 28)

**Included**
