# Supplementary material for: What are the experiences of people with heart failure regarding participation in physical activity? A systematic review, meta-aggregation and development of a logic model
Source: BMJ Open. 2025 Apr 5;15(4):e092457. doi: 10.1136/bmjopen-2024-092457 (PMC11973767; doi:10.1136/bmjopen-2024-092457)
Supplement: online supplemental file 3 [file bmjopen-15-4-s003.docx]

**Appendix 3 : Reporting of Progress-plus items of the papers that included data from people with heart failure.***

| **Progress Plus items** | **Reporting by % paper and detail** |
| --- | --- |
| **Place of residence** | **93% (26/27)**   - 1 study recruited hospital patients; the rest were home-dwelling . |
| **Race/ethnicity/culture/language** | - **59% (16/27)** - 10/16 papers detailed ethnicity. - 2 UK papers reported 100% White & 1 further UK paper reported 69% White, 6% Pakistani, 6% Irish white , 6% other White & 12% Asian. - 1 Australian paper 88% White, 12% other. - 6 USA papers 31-91% White, 0-50% black, 0-29% Hispanic, 0-15% Asian and 0-14% ‘other’ - Language requirements were in 10/16 papers, most commonly English. |
| **Occupation** | **26% (7/27)**   - Retirement was the most common occupation in these papers. |
| **Gender/sex** | **93% (25/27)**   - One study recruited only men, and one study recruited only women - 68% (17/25) papers which described mixed gender/sex recruitment includes ≤50% women participants, often significantly so. |
| **Religion** | **0% (0/27)** not reported in any paper |
| **Education** | **33% (9/27)**   - A range of educational achievement from secondary school to higher education. |
| **Socioeconomic status (SES)** | **7% (2/27)**   - One paper described household income and another paper subjective SES. - Three other papers described the need to have the internet, technology and space to be involved in the intervention. |
| **Social capital** | **26% (7/27)**   - These papers described being married/cohabiting or living with other people. |
| **Personal characteristics associated with discrimination** | **78% (21/27**) described NYHA status, with NYHA II & III the most common status.   - Class IV patients were excluded in 7 studies and class II patients excluded in 2 studies and class I patients excluded in 3 studies   **81% (22/27)** gave mean/median age   - Participant age range across all papers were 34-94 years - Only 41% (9/22) papers detailed mean ages of ≥70 years ( average diagnosis in the UK is 76 years)   One study, required participants to have ‘high levels of technology, symptom and HF knowledge’  NINE studies excluded on COGNITIVE IMPAIRMENT.  Other studies had criteria such as KNOWLEDGE REQUIREMENTS (1 study), CAPACITY TO CONSENT (one study), or excluded participants with ‘OTHER SERIOUS PHYSICAL OR MENTAL DISORDERS (one study) and PSYCHIATRIC DISORDERS (5 studies). |
| **Features of relationships** | **0% (0/27)**   - Nothing relevant to report |
| **Time-dependent relationships** | **41% (11/27)**   - These were recruited on outpatients/within hospital & therefore were likely to have had hospital /tertiary care recently |

* Progress-plus items of papers are presented that describe characteristics around people with heart failure (27 papers). We have not included health professional data (one study) and carer data (two studies) in this table as it was limited. The three additional papers associated with two intervention studies provided different profiles of people with heart failure and their characteristics, so data is presented predominantly by paper content, not study content.
