# Supplementary material for: What are the experiences of people with heart failure regarding participation in physical activity? A systematic review, meta-aggregation and development of a logic model
Source: BMJ Open. 2025 Apr 5;15(4):e092457. doi: 10.1136/bmjopen-2024-092457 (PMC11973767; doi:10.1136/bmjopen-2024-092457)
Supplement: online supplemental file 4 [file bmjopen-15-4-s004.docx]

**Appendix 4: JBI checklist for qualitative studies.**

| **Study** | **1. Congruity between stated philosophical perspective and research methodology?** | **2. Congruity between research methodology and research question or objectives?** | **3.Congruity between research methodology and data collection methods?** | **4. Congruity between research methodology and representation and analysis of data?** | **5. Congruity between research methodology and interpretation of results?** | **6. Statement locating the researcher culturally or theoretically?** | **7. Influence of the researcher on the research, and vice- versa, addressed?** | **8. Are participants, and their voices, adequately represented?** | **9a. Is the research ethical according to current criteria or, for recent studies?** | **9b. Is there evidence of ethical approval by an appropriate body?** | **10. Do the conclusions drawn in the research report flow from the analysis, or interpretation, of the data?** |
| --- | --- | --- | --- | --- | --- | --- | --- | --- | --- | --- | --- |
| Albert 2015 | YES | YES | YES | YES | YES | NO | NO | NO | YES | YES | YES |
| Amirova 2022 | YES | YES | YES | YES | YES | NO | NO | YES | YES | YES | YES |
| Cewers 2019 | YES | YES | YES | YES | YES | YES | NO | YES | YES | YES | YES |
| Charuel 2022 | YES | YES | YES | YES | YES | NO | NO | YES | YES | YES | YES |
| Durante 2018 | YES | YES | YES | YES | YES | YES | YES | Unclear | YES | YES | YES |
| Eckerblad 2023 | YES | YES | YES | YES | YES | YES | YES | YES | YES | YES | YES |
| Europe2004 | YES | YES | YES | YES | YES | NO | NO | NO | YES | YES | YES |
| Markus 2023 | YES | YES | YES | YES | YES | YES | YES | YES | YES | YES | YES |
| Meeker 2019 | YES | YES | YES | YES | YES | NO | YES | NO | YES | YES | YES |
| Nikasson 2022 | YES | YES | YES | YES | YES | YES | Unclear | YES | YES | YES | YES |
| Pihl 2011 | YES | YES | YES | NO | NO | NO | NO | YES | YES | YES | YES |
| Saifan 2024 | YES | YES | YES | YES | YES | NO | YES | YES | YES | YES | YES |
| Tierney 2011 | YES | YES | YES | YES | YES | NO | YES | YES | YES | YES | YES |
| Walthall 2019 | YES | YES | YES | YES | YES | NO | YES | YES | YES | YES | YES |
| Adsett 2019 | NO | YES | YES | YES | YES | NO | YES | NO | YES | YES | YES |
| Barlett 2014 | YES | YES | YES | YES | YES | NO | NO | YES | YES | YES | YES |
| Hägglund 2018 | YES | YES | YES | NO | YES | NO | NO | NO | Unclear | YES | YES |
| Hwang 2017 | YES | YES | YES | YES | YES | NO | YES | YES | YES | YES | YES |
| Macagal 2021 | YES | YES | YES | YES | YES | NO | NO | YES | YES | YES | YES |
| Okwose 2020 | YES | YES | YES | YES | YES | YES | YES | YES | YES | NO | YES |
| Selman 2015 | YES | YES | YES | YES | YES | NO | NO | YES | YES | YES | YES |
| Warehime 2020 | YES | YES | YES | Unclear | YES | Unclear | YES | Unclear | YES | YES | YES |
| Yeh 2016 | YES | YES | YES | YES | YES | YES | YES | NO | YES | YES | YES |
| Cacciata 2021 | NO | YES | YES | YES | YES | NO | Unclear | NO | YES | YES | YES |
| Klompstra 2017 | NO | YES | YES | YES | YES | NO | YES | Unclear | YES | YES | YES |
| Klompstra 2021 | NO | YES | YES | YES | YES | NO | YES | NO | Unclear | YES | YES |
| Frost 2019 | YES | YES | YES | YES | YES | NO | NO | Unclear | YES | YES | Unclear |
| Smith 2021 | YES | YES | YES | YES | YES | NO | NO | YES | YES | YES | YES |
