# Supplementary material for: What are the experiences of people with heart failure regarding participation in physical activity? A systematic review, meta-aggregation and development of a logic model
Source: BMJ Open. 2025 Apr 5;15(4):e092457. doi: 10.1136/bmjopen-2024-092457 (PMC11973767; doi:10.1136/bmjopen-2024-092457)
Supplement: Supplementary file 5 [file bmjopen-2024-092457.reviewer_comments.pdf]

PEER REVIEW HISTORY

BMJ Open publishes all reviews undertaken for accepted manuscripts. Reviewers are asked to complete a checklist review form and are provided with free text boxes to elaborate on their assessment. These free text comments are reproduced below.

ARTICLE DETAILS

Title (Provisional)

What are the experiences of people with heart failure regarding participation in physical activity? A systematic review, meta-aggregation and development of a logic model.

Authors

Huntley, Alyson; Duncan, Lorna; Essery, Rosie; Dawson, Shoba; Ismail, Yasmin; Baird, Justine; Butcher, Karen; Whight, Emily; Johnson, Rachel

---

VERSION 1 - REVIEW

Reviewer 1

Name Tierney, Stephanie

Affiliation University of Oxford, Nuffield Department of Primary Care Health Sciences

Date 23-Sep-2024

COI I have previously published with Dr Shoba Dawson and I am currently working on studies with her about social prescribing.

This is an interesting and well-presented qualitative systematic review that was used to inform the development of a logic model. I just have a few minor points for consideration by the authors.

1. Perhaps outline stages associated with meta-aggregation a bit more for those unfamiliar with it. Perhaps give background to meta-aggregation in terms of being focused on the development of statements to guide practitioners and policy makers.

2. Logic model development described in the methods – how many stakeholders were involved in this? Did it involve a specific meeting or separate conversations with individuals?

3. I was concerned about the use of the term 'risk of bias' in appendix 4 – as the idea of bias in qualitative research is a contested term – see work by Braun and Clarke relating to embracing the idea of subjectivity in qualitative research.

4. Figure 2 – the logic model was hard to read because the writing was so small.

5. I wondered why statement 5 had been divided into a and b rather than being written as 2 separate statements? Are you saying that intrinsic motivation would be upheld by support from others or could be affected by outside influences?

6. In the discussion, I wasn't quite sure what was meant when the authors write 'On the basis of the study's findings…' (page 16, line 36) – can this be made clearer?

Reviewer 2

Name Forsyth, Faye

Affiliation University of Cambridge School of Clinical Medicine, Public Health and Primary Care

Date 30-Oct-2024

COI None

Thank you for the opportunity to review this important piece of work, which is very timely given the publication of a few landmarks studies in the last five years.

General comment: This is quite a large piece of work and I wonder whether it would benefit from being separated to enable a fuller and more detailed report (i.e. 1 paper with the meta-aggregation, one with the themes mapped to COM-B/TDF and the LM, a possible third paper with the EDI assessment?

Abstract: should it be 'for a future intervention' or 'future interventions'

Background - the first statement requires a reference.

Paragraph 2 opening sentence does not seem grammatically correct and the link between paragraph one and 2 seems incomplete, consider revisiting. Second sentence in paragraph 2 needs a reference.

Line 40 - should it be 'issues' not 'issue'

General comment: There are quite a few unusual grammatical, syntax and presentation inconsistencies, the paper may benefit from a robust re-read/editing.

Search strategy - was there justification for the databases? The absence of CINAHL and AMED might be a relevant limitation as both nurses and physiotherapists often lead PA related work.

Eligibility - it is important to describe the phenotypes of heart failure included, does this review include HFrEF, HFmrEF, HFpEF? Was this a consideration as data in HFpEF suggests different pathways for breathlessness/fatigue for HFpEF than for HFrEF (fat infiltration in muscle and muscle oxygen extraction/perfusion problems). Perhaps setting out the PICO criteria would help readers understand the breadth of the work better?

If you considered any type of PA, then it would seem reasonable to include studies that focussed on self-care, of which PA is an important component? Later you state that you included studies that described perceptions of HF patients living with their illness, if this is the case then a large body of studies would seem to be missing from the review? (see table added as attachment)

The synthesised findings expressed as statements lack deep description, likely due to the volume of work that the authors have presented within this report, therefore it is difficult to really understand the substance/depth/breadth behind these statements. Some particular comments are presented below:

Results 1: Physical symptoms. It is not clear whether this synthetises construct is limited to heart failure symptoms, it would seem that it is not? This should be expanded to clarify is possible, most patients with HF will have multi-morbidity (quote 1) which may explain a lot of symptomology?

Results 2: Emotional Factors. What symptoms are people fearful of exacerbating, breathlessness, pain, MSK issues? All quotes related to fear, it is difficult to verify the 'motivation', could this be added as an alternative? The quote presented in theme 3 extrinsic factors about 'being overwhelmed' hints that emotional factors could be more than just fear of worsening symptoms, it seems like psychological factors such as self-efficacy and resilience might also be at play?

Result 3: Extrinsic factors. Were other factors like the beliefs of others not important here? Quite a lot of literature on activity in heart failure states that the beliefs of others can modify engagement both positively and negative (i.e. Tierney and Smith) Is the quote on asthma more about their co-existing conditions rather than the weather i.e. concurrent asthma modifies their response to the weather, rather than the weather per se - perhaps this belongs in the physical symptoms section?

Result 4. note typo 'reenforces' should be reinforces. Is this also about safety and not just knowledge?

Result 5. As above, does this not overlap with extrinsic factors?

Result 6. Are the experiences of pain, fear and breathlessness reported earlier not also negative responses to activity? There are also quotes to suggest that being worse off financially is a potential negative consequence?

Discussion:

Overall the discussion is quite short and with the exception of the comparison to the Cochrane cardiac rehab review, it does not compare the findings to other studies, should this be expanded?

The review does not include cardiac rehabilitation studies, which is open to people with HF. Cardiac rehab has a long history of offering remote and digital physical activity via structures

rehabilitation pathways, if these studies were included, would it change conclusions relating to technological/digital abilities?

The conclusions about embedding support in the community does not link obviously (at least not to me), from the findings of the review (synthesised statements). Whilst some activity is better than none, it is not clear that 'PA in everyday life', is sufficient to improve symptoms and quality of life in HF? Current evidence suggests that HIIT and continuous training are more optimal approaches for improving cardiorespiratory fitness, are these approaches compatible with 'PA in everyday life'? Future interventions need to be cognisant of the many barriers as highlighted, but they also need to acknowledge the clinical evidence base which highlights these approaches.

Overall this is a very large piece of work and the authors should be congratulated for the robust approaches they have adopted. However, I feel that presenting everything within the one report limits the readers' ability to appreciate the breadth and depth of the synthesised statements, as they are minimally described in order to allow for the integration of the other components of the review within in word limit of the journal.

Primary Qualitative Studies Exploring the HF Experience based on recent reviews

REVIEW	Author	Year	Title	Inclusion Criteria ± Sample HFpEF/HFrEF Characteristics	Sample Category: HFrEF, HFpEF, Mixed HF, HF Unspecified
Austin et al 2021	Ahmad et al.	2016	Comparing Perspectives of Patients, Caregivers, and Clinicians on Heart Failure Management	Patients were eligible for this study if they were admitted to a cardiology or general medicine service at either site with a primary diagnosis related to HF. Heart failure patients were identified by a combination of an electronic health record query that generated a daily census of patients admitted with a HF or HF-related diagnosis, chart review by research assistants, and direct contact with floor nurses.	HF Unspecified
	Allen et al.	2009	The Lived Experience of Middle-Aged Women with New York Heart Association Class III Heart Failure: A Pilot Study	Potential participants were screened for inclusion by a HF case manager employed in a university affiliated cardiology practice using the following criteria. Participants had to be: (1) female, (2) diagnosed with NYHA class III HF, (3) at least 21 years of age, (4) able to verbally articulate their experience with HF, and (5) willing to participate	HF Unspecified
	Andersson et al.	2012	Living with heart failure without realising: a qualitative patient study	Medical record system search to identity those with ICD10 codes for HF.	HF Unspecified
	Chiaranai et al.	2014	A Phenomenological Study of Day-to-Day Experiences of Living With Heart Failure	Fifteen Thai men and women with HF in New York Heart Association classes I to III, aged between 47 and 75 years, were interviewed with open-ended questions.	HF Unspecified
	Cortis and Williams	2007	Palliative and supportive needs of older adults with heart failure	Patients attending an Heart Failure Support Program were approached. Initial eligibility criteria for the study included all those patients currently attending the support programme who were deemed by their HFNS to be well enough to be approached.	HF Unspecified
	Falk et al.	2007	Keeping the maintenance of daily life in spite of Chronic Heart Failure. A qualitative study	Patients attending a heart failure out-patient clinic, nurse led, for regular review were invited to participate. The informants' HF was classified according to New York Heart Association [NYHA] functional classification III and IV	HF Unspecified
	Fry et al.	2016	The implications of living with heart failure; the impact on everyday life, family support,	participants who had been identified and recruited for the HoldFAST study from a cardiology ward and specialist heart failure ambulatory clinic from three geographical locations within the UK; the Midlands, South Central	HF Unspecified

			co-morbidities and access to healthcare: a secondary qualitative analysis	and the South West of England. Patients with severe or difficult to manage heart failure from the Midlands and South Central and less 'severe' patients from a primary care centre from the South West of England were approached.	
	Gallacher et al.	2011	Understanding Patients' Experiences of Treatment Burden in Chronic Heart Failure Using Normalization Process Theory	All patients had chronic heart failure confirmed by echocardiography and were receiving treatment with both angiotensin-converting enzyme inhibitors and diuretics from their family practitioner.	HF Unspecified
	Gwaltney et al.	2012	Hearing the voice of the heart failure patient: key experiences identified in qualitative interviews	Interview with chronic heart failure patients, parsed into HFrEF (30%), HFpEF (65%) and HFmrEF (5%). (HFpEF n=26)	Mixed
	Hoe et al.	2019	Patients' Beliefs About Causes and Consequences of Heart Failure Symptoms	The inclusion criteria were confirmed diagnosis of HF (medical record), New York Heart Association functional Classes II to IV, ability to read and speak English,	HF Unspecified
	Holden et al.	2015	The patient work system: An analysis of self-care performance barriers among elderly heart failure patients and their informal caregivers	Data were collected from and about elderly heart failure patients (N=30) and their informal caregivers (N=14) using research interviews, observations, surveys, and medical record review	HF Unspecified
	Hopp et al.	2012	Life Disruption, Life Continuation: Contrasting Themes in the Lives of African-American Elders With Advanced Heart Failure	Participants included 35 African Americans aged 60 and over with advanced heart failure, identified through records from a community hospital in Detroit, Michigan	HF Unspecified
	Kimani et al.	2018	Multidimensional needs of patients living and dying with heart failure in Kenya: a serial interview study	We purposively recruited 18 patients admitted with advanced heart failure at a rural district hospital in Kenya.	HF Unspecified
	Mahoney-Davies et al.	2017	A Thematic Analysis of Emotional and Psychological Experiences of People with Heart Failure	A targeted recruitment approach was used to identify people who would be able to describe a breadth of experience.	HF Unspecified
	Malhotra et al.	2016	Living with heart failure: Perspectives of patients from Singapore	Clinical coordinators identified and screened patients with heart failure admit-ted to the hospital's cardiology department from the clinical system on a daily basis. Eligible participants were at least 18 years old, had heart failure and were judged by the clinical coordinators to be physically and mentally competent to participate.	HF Unspecified
	Mangolian shahrbabaki et al.	2017	The sliding context of health: the challenges faced by patients with heart failure from the perspective of patients, healthcare providers and family members	A total of 21 participants were selected using purposive sampling strategy by the first author from cardiovascular departments of the educational hospitals of Kerman, which are the largest referral centres of patients in the south-east of Iran.	HF Unspecified
	Ming et al.	2011	Perspectives of heart failure patients in Malaysia towards medications and disease state management: findings from a qualitative study	Purposive sample of 20 patients on the basis of their age, sex, ethnicity, and region of residence. Selection criteria included confirmed diagnosis of CHF by a cardiologist; readmission to the cardiology ward in Penang General Hospital for HF within the past 90 days; ability to read and speak in the English, Malay, or Chinese language; and alert and oriented with no coexisting terminal illnesses.	HF Unspecified
	Moshki et al.	2019	Dark or Bright Half of the Moon: A Qualitative Study Exploring the Experience of Iranian Heart Failure Patients Regarding their Quality of Life	Out of the 19 participants in this study, 14 patients were admitted to Imam Reza Hospital, consisting of those who had referred to the HF subspecialty clinic. The other 5 participants had referred to a specialised HF outpatient private clinic in Mashhad, Iran. The mean age (SD) of the participants was 59.5 (12.4) years, and the mean duration of the disease was 5.2 years. The mean (SD) of ejection fraction was 23.1% (8.3).	HFrEF
	Nordfinn et al	2019	Patients' experience with heart failure treatment and self-care—A qualitative study	Eligible patients were identified by screening the medical records from the patient list at the nurse-led HF outpatient clinic emphasising the specialist nurses' latest journal note and registered New York Heart Association	HF Unspecified

			exploring the burden of treatment	(NYHA) class. The inclusion criteria were diagnosis of HF confirmed by echocardiography at least three months prior and NYHA class II or III.	
	Nordgren et al.	2007	Living with moderate-severe chronic heart failure as a middle-aged person	Inclusion criteria were a history and diagnosis of moderate-to-severe CHF (NYHA III-IV1); and a minimum of one hospitalization due to CHF during the previous year.	HF Unspecified
	Paton et al.	2007	Recalibrating time and space: women's challenges of living with heart failure	The researchers of this interpretive research encouraged the seven women who volunteered for this study to talk about their day-to-day challenges	HF Unspecified
	Pattenden et al.	2007	Living with heart failure; patient and carer perspectives	Employed purposive sampling based on the theoretical principle that it is good practice to ensure the maximum variation of patients and settings. The five categories used in the sampling strategy were: gender, age, ethnic group, NYHA classification of severity of heart failure and centre. Seven or eight patients from each centre were sampled from the total of 124 who agreed to be interviewed.	HF Unspecified
	Paturzo et al.	2016	The lived experience heart failure: a phenomenological study	Meeting ESC HF criteria	HF Unspecified
	Piamjariyakul et al.	2012	Part I: Heart failure home management: Patients, multidisciplinary health care professionals and family caregivers' perspectives	Inclusion criteria were the patients needed to be alert and oriented, as assessed by a nurse practitioner; able to read and speak English; have a diagnosis of systolic HF; and 1 year prior enrolment in a multidisciplinary HF hospital discharge education-based program, based on national HF guideline	HFrEF
	Rerkluenrit et al.	2009	Self-Care among Thai People with Heart Failure	Theselectioncriteriaincludedindividualswhowere:1) diagnosed with heart failure by a cardiologist; 2) over 18 years old; and, 3) born, raised and presently living in Thailand.	HF Unspecified
	Retrum et al.	2013	Patient-Identified Factors Related to Heart Failure Readmissions	Patients were recruited from an academic referral hospital and a community-based hospital. Patients discharged with a primary discharge diagnosis for HF who were then readmitted for any cause in the subsequent 180 days were eligible for the study	HF Unspecified
	Ryan and Farrelly	2009	Living with an unfixable heart: a qualitative study exploring the experience of living with advanced heart failure	purposive sampling was used to subjectively select participants considered likely to own rich experiential accounts regarding the experience of living with heart failure. Seventeen people attending the hospital cardiology outpatient clinic were invited to participate.	HF Unspecified
	Sano and Majima	2018	Self-management of congestive heart failure among elderly men in Japan	Eligible subjects were recruited from one Japanese hospital with cardiovascular outpatient clinic services. Medical records were used to screen the population for eligible persons. The subject inclusion criteria were (1) ≧65 years of age, (2) no re-hospitalizations for >2 years since the last hospitalization, (3) cardiac status ranked as New York Heart Association (NYHA) class 2 or 3, and (4) the ability to converse in Japanese. (HFpEF n=1)	Mixed
	Seah et al.	2016	Experiences of Patients Living With Heart Failure: A Descriptive Qualitative Study	A purposive sample of 15 informants was recruited from two cardiology wards of a tertiary public hospital in Singapore.	HF Unspecified
	Tenner	2018	Patient Perspective: LIVING WITH HEART FAILURE Everybody Has a Story, and I Am Lucky!	NA – patient letter. HFpEF (n=1)	HFpEF
	Walthall et al.	2017	Living with breathlessness in chronic heart failure: a qualitative study	Twenty-five participants with heart failure with reduced ejection fraction (HFrEF) from a tertiary referral centre in England were recruited	HFrEF
	Walthall et al.	2019	Patients experience of fatigue in advanced heart failure	Inclusion criteria: 18 years and older, diagnosis of New York Heart Association class III-IV heart failure with reduced ejection fraction and able to participate in interviews in their own language	HFrEF
	Woda ete al.	2015	Factors influencing self-care behaviours of African Americans with heart failure: A photovoice project	Case managers reviewed the onsite health records to identify individuals with a history of HF and discussed the study with these potential participants. The case manager provided names and contact information of interested residents to the primary investigator (PI) who visited residents in their apartments to explain the study. Those who wanted to participate completed the informed consent and demographic questionnaires during this visit. Ten residents participated.	HF Unspecified

Jeon et al. 2010	Davidson et al.	2007	Cultural diversity In heart failure management: Findings from the Discover Study (Part 2)	Thirteen family groups were interviewed for the study with a representation across the identified cultural groups.	HF Unspecified
	Costello et al.	2004	What is the experience of men and women with congestive heart failure?	This qualitative case study approach used semi structured interviews with women and men with New York Heart Association (NYHA) class three or four CHF who were referred to a CHF clinic.	HF Unspecified
	Simpson et al.	2000	Using focus groups to identify barriers to drug use in patients with congestive heart failure	Participants in the first three focus groups were patients attending a heart failure clinic. The clinic is managed by a multidisciplinary team of physicians, specialized nurse practitioners, and pharmacists to provide an optimum level of wellness for ambulatory patients with CHF.	HF Unspecified
	Buetow et al.	2001	Coping strategies in the self-management of chronic heart failure	Patients >45 years of age with a record of HF. Diastolic HF patients excluded	HFrEF
	Bränstrom et al.	2006	Living with severe chronic heart failure in palliative advanced home care	Patients with severe CHF, one woman (NYHA III) and three men(NYHAIV),participated in the study. Three patients had a confirmed diagnosis in accordance with the criteria proposed by the European Society of Cardiology.	HF Unspecified
	Ekman et al	1999	The meaning of hospital care, as narrated by elderly patients with chronic heart failure	12 elderly patients with severe chronic heart failure	HF Unspecified
Jeon et al. 2010	Ekman et al.	2001	A place of ones' own. The meaning of lived experience as narrated by an elderly woman with severe chronic heart failure. A case-study	1 patient with severe heart failure	HF Unspecified
	Eldh et al.	2006	The meaning of patient participation for patients and nurses at a nurse-led clinic for chronic heart failure.	The severity of the patients' HF was classified as New York Heart Association (NYHA) classes II and III. The patients met the criteria of being able to give informed consent and to communicate in Swedish	HF Unspecified
	Europe et al.	2004	Qualitative analysis of the male experience of heart failure	Twenty men, aged between 43 and 73 years, with moderate chronic HF (Table I) gave their informed consent to participate in the present study, which was approved by the local ethics committee.	HFrEF
	Hagglund et al.	2008	The experience of fatigue among elderly women with chronic heart failure	The inclusion criteria were: female gender, an age of 70 years or older, a diagnosis of CHF confirmed by the criteria of the European Society of Cardiology, and the ability to communicate in Swedish	HF Unspecified
	Martensson et al.	1997	Male patients with congestive heart failure and their conception of the life situation	The informants were asked to participate during a visit to the medical clinic of a county hospital situated in the south-western part of Sweden.	HF Unspecified
	Martensson et al.	1998	Female patients with congestive heart failure: how they conceive their life situation	The informants were asked to participate during a visit to the medical clinic of a county hospital situated in the south-western part of Sweden.	HF Unspecified
	Bennett et al.	2000	Self-Care Strategies for Symptom Management in Patients With Chronic Heart Failure	All patients enrolled in the veterans centre had previously documented evidence of chronic heart failure by radiologic or echocardiographic examination. Names of potential participants enrolled in the GMC were obtained from the computerized medical records database associated with this facility and based on the following echocardiographic findings indicative of heart failure within the past 6 months: (a) left ventricular internal diastolic dimension of 5.5 or greater, (b) fraction of systolic shortening of 18% or less, (c) left ventricular ejection fraction of 40% or less, or (d) generalized abnormal ventricular wall motion abnormality.	HFrEF
	Bentley et al.	2005	Factors related to nonadherence to low sodium diet recommendations in heart failure patients	Using convenience sampling, 20 patients who met the following criteria were enrolled in this study: 1) diagnosis of HF due to left ventricular systolic dysfunction with ejection fraction less than 50%, 2) healthcare provider's recommendation to follow a low sodium diet, 3) no cognitive impairments limiting the ability to complete an interview, 4) not living in an extended care facility, and 5) on stable medical therapy without major recent adjustments	HFrEF

	Bosworth et al.	2004	Congestive heart failure patients' perceptions of quality of life: the integration of physical and psychosocial factors	We convened three focus groups with 15 male CHF patients. Eligible individuals had a documented outpatient diagnosis of CHF (ICD-9 codes 428, 402.01, 402.11, 402.91, 425.4), visited the general medicine clinic of the Durham VAMC in 1999 at least once, and had an ejection fraction of less than 40%.	HFrEF
	Chang et al.	2004	Relaxation response for Veterans Affairs patients with congestive heart failure: results from a qualitative study within a clinical trial	We enrolled a total of 95 ambulatory chronic HF patients with moderate levels of symptom severity (NYHA class II or III) and reduced left ventricular ejection function (≤40%).	HFrEF
	Evangelista et al.	2001	Gender differences in health perceptions and meaning in persons living with heart failure	The convenience sample included 32 persons (50% women) with a primary diagnosis of HF who were receiving care at a single outpatient HF clinic. Patients were included in the study if they were alert and oriented, able to understand English, and they agreed to give informed consent. Descriptive statistics indicate all were HFrEF: Ejection fraction (mean ± SD) 26.69 ± 11.46	HFrEF
	Mahoney et al.	2001	An ethnographic approach to understanding the illness experiences of patients with congestive heart failure and their family members	Physicians and nurses were asked to identify patients who could richly describe their experience. Parameters for criterion sampling included ethnically diverse, English speaking, male and female medically diagnosed and medically treated patients with CHF who had not had coronary artery bypass surgery in the last 5 years, who were 50 years of age or older, and whose English-speaking family members were 12 years of age or older	HF Unspecified
	Miller	2000	Cue sensitivity in women with cardiac disease	Interviews included 10 women with cardiac disease, two women at risk for cardiac disease, three advanced nurse practitioners, and one cardiologist. Participants were interviewed on two occasions.	HF Unspecified
Jeon et al. 2010	Rhodes et al.	2002	Heart failure and its impact on older women's lives	Inclusion criteria for participants included that they were women ranging in age from 60 to 90 years who self-reported that they had been diagnosed by their cardiologists with The New York Heart Association (NYHA) Stage II HF. If the participant was uncertain of her HF Stage, the researcher determined the stage through a limited physical examination and questions regarding activities of daily living.	HF Unspecified
	Riegel and Carlson	2002	Facilitators and barriers to heart failure self-care	Previous hospitalisation due to CHF. Diagnosis confirmed in medical records.	HF Unspecified
	Rucker-Whitaker et al.	2006	Understanding African-American participation in a behavioral intervention: Results from focus groups	Patients recruited from he Heart Failure Adherence and Retention Trial (HART) is the largest NHLBI funded behavioral clinical trial of patients with HF (R01 HL65547). HART is following 900 mild to moderate HF patients over an average of 3 years. 80% in original trial had an EF<40%	Mixed HF
	Scotto	2005	The Lived Experience of Adherence for Patients With Heart Failure	Fourteen patients attending an outpatient heart failure clinic after hospital readmission for exacerbation of heart failure symptoms were interviewed	HF Unspecified
	Stull et al.	1999	Becoming a patient with heart failure	Twenty-one patients with a diagnosis of heart failure were conveniently selected and interviewed for this research. Patients were selected on the basis of having a left ventricular ejection fraction of 35% or less and New York Heart Association class II, III, or IV.	HFrEF
	Westlake e tal.	2001	Role of Spirituality in Adjustment of Patients with Advanced Heart Failure	Purposive sampling of all patients who presented to two university-affiliated, outpatient heart failure clinics was used.	HF Unspecified
	Zambroski	2003	Qualitative analysis of living with heart failure	Inclusion criteria included the following: patients were (1) diagnosed with heart failure, (2) undergoing medical treatment for heart failure, (3) English speaking, and (4) 40 years of age or older. Participants were recruited from a local hospital, a visiting nurse association, a mall health center, and professional referral.	HF Unspecified
Welstand et al. 2009	Dunnerdale et al.	2007	Health-related quality of life from the perspective of patients with chronic heart failure	The sample consisted of 11 patients admitted to a district general hospital with an objective medical diagnosis of CHF between October 2003 and February 2004. All had LVSD of varying severity	HFrEF
	Thornhill et al	2008	Experiences of living with congestive heart failure: A qualitative study	Twenty-five patients were recruited from City Hospital, Birmingham, UK. Thirteen were out-patients and twelve in-patients. Out-patients were selected from the out-patients heart clinic or from a programme for the rehabilitation of heart failure patients. Participants were selected for diversity of ages, backgrounds and history of CHF in order to obtain r ich	HF Unspecified

				data to explore themes and patterns within different experiences of living with heart failure, rather than focusing on demographics	
Falk et al. 2013	Barnes et al.	2006	Communication in heart failure: perspectives from older people and primary care professionals	The inclusion criteria were that participants must be over 60 years of age and have a New York Heart Association (NYHA) classification of III or IV heart failure	HF Unspecified
	Gott et al.	2008	Older people's views of a good death in heart failure: implications for palliative care provision	Patients were considered eligible for recruitment into the study if they were>60 years, could speak English, did not have evidence of significant cognitive impairment, and had self-reported New York Heart Association Classification(NYHA) class II–I	HF Unspecified
Primary qualitative studies exploring the experience of HF, collated from five systematic reviews, duplicates, studies with professionals and quantitative studies removed. The systematic review by Yu et al. was reviewed for studies however all studies in this review had been included by the previous reviews.					

**Reviewer**	**3**
**Name**	**Brown Wilson, Christine**
**Affiliation**	**Queen's University Belfast**
**Date**	**20-Jan-2025**
**COI**	**None**

Thank you for the opportunity to review this paper that outlines the development of a logic model from a systematic review and met-aggregation of qualitative data alongside a study within a review process for consideration of EDI factors. The involvement of a PPI group in this process is a strength of this work and well documented for others to follow. The only suggestion I would make would be to explain the process of meta aggregation as opposed to a general synthesis of the data. Overall the process leading to the findings is well documented with clear tables. The data presented in the findings underpin each theme with a clear link to the logic model, which has also been presented for a non academic audience. The discussion clearly outlines the issues that have been raised with clear recommendations for future studies. I hope you find these comments helpful.

VERSION 1 - AUTHOR RESPONSE

Responses to Reviewer: 1 Dr Stephanie Tierney, University of Oxford

1) Perhaps outline stages associated with meta-aggregation a bit more for those unfamiliar with it. Perhaps give background to meta-aggregation in terms of being focused on the development of statements to guide practitioners and policy makers.

Agree, this would be useful. We have added background and more detailed explanation of the three main stages of meta-aggregation within a figure to ensure we keep within the word count.

2) Logic model development described in the methods – how many stakeholders were involved in this? Did it involve a specific meeting or separate conversations with individuals?

   We have put more detail of the clinical co-investigators and other collaborators in the methods along with the existing detail of the PPI group. We have also added detail of both groups contribution under the logic model methods section

3) I was concerned about the use of the term 'risk of bias' in appendix 4 – as the idea of bias in qualitative research is a contested term – see work by Braun and Clarke relating to embracing the idea of subjectivity in qualitative research.

   This appendix label has been revised and now reads 'JBI checklist for qualitative studies.'

4) Figure 2 – the logic model was hard to read because the writing was so small.
   We agree. The problem is that the working logic model is too detailed to really fit onto an A4 side. We have produced a simpler version for publication but have cited that the full and PPI version of the logic model are available upon request from the authors should the readers want to know more. This means we do not have excessive amounts of figures and tables with the publication and keeps the focus on the systematic review and meta-aggregation.

5) I wondered why statement 5 had been divided into a and b rather than being written as 2 separate statements? Are you saying that intrinsic motivation would be upheld by support from others or could be affected by outside influences?

When composing these two synthesised findings we discussed having these two separate and together. This was based on the interconnected content of the synthesis around these two points. With further team discussion we have decided to separate these synthesised findings to ensure their meaning is clear and they are indeed two different findings, but we have left

the supporting text and quotes together to highlight that they are closely connected.

6) In the discussion, I wasn’t quite sure what was meant when the authors write ‘On the basis of the study’s findings…’ (page 16, line 36) – can this be made clearer?

This has been revised. We agree !, It did not make sense.

Response to Reviewer: 2 Mrs. Faye Forsyth, University of Cambridge School of Clinical Medicine

Thank you for the opportunity to review this important piece of work, which is very timely given the publication of a few landmarks studies in the last five years.

7) General comment: This is quite a large piece of work, and I wonder whether it would benefit from being separated to enable a fuller and more detailed report (i.e. 1 paper with the meta-aggregation, one with the themes mapped to COM-B/TDF and the LM, a possible third paper with the EDI assessment?

Please see the general statement at the beginning of our responses.

8) Abstract: should it be 'for a future intervention' or 'future interventions'

This has been revised.

9) Background - the first statement requires a reference.

This has been revised.

10) Paragraph 2 opening sentence does not seem grammatically correct and the link between paragraph one and 2 seems incomplete, consider revisiting.

This has been revised.

18) Second sentence in paragraph 2 needs a reference.

This has been revised.

19) Line 40 - should it be 'issues' not 'issue'

This has been revised.

AS comment 4) General comment: There are quite a few unusual grammatical, syntax and presentation inconsistencies, the paper may benefit from a robust re-read/editing.

Response as comment 4

20) Search strategy - was there justification for the databases? The absence of CINAHL and AMED might be a relevant limitation as both nurses and physiotherapists often lead PA related work.

This was discussed as a team and the decision was to go with the three databases cited as we felt that they would capture 99% plus of our desired studies. This was based on our previous experience in searching for heart failure studies. We decided to use Ovid Emcare database to cover nursing, physiotherapy and other allied health profession research. This was in part a new strategy for team as we have previously used CINAHL for nursing studies. We were aware that the indexing and ease of use Emcare (OVID) was likely to be better than CINHAL, which falls down on both these points. Our final inclusion of papers and after the forward and backward referencing list checking we felt that we made a good choice. We acknowledge searching is not the exact science we would all like, but we are keen to be pragmatic in our choices. In this instance we did not feel that AMED was a useful choice for our topic area. Our previous experience with AMED is that is a limited database and has added little to nothing in previous reviews.

We have added brief descriptors after the database name in the methods to reflect why we used them.

21) Eligibility - it is important to describe the phenotypes of heart failure included, does this review include HFrEF, HFmrEF, HFpEF? Was this a consideration as data in HFpEF suggests different pathways for breathlessness/fatigue for HFpEF than for HFrEF (fat infiltration in muscle and

muscle oxygen extraction/perfusion problems). Perhaps setting out the PICO criteria would help readers understand the breadth of the work better?

We agree this important in many heart failure studies but in this review firstly we were interested in the personal experiences of people with any phenotype of heart failure. As with any condition people cope very differently with the same diagnosis/phenotype.

Secondly most qualitative studies also reflect that and give modest/general inclusion criteria. Of course, the clinical studies were more explicit (see table 1).

Most of the included studies focused on chronic heart with reduced ejection fracture, some of them with more detail than others, Only two studies recruited People with HFpEF. Adsett recruited both reduced and preserved EF participants and Smith was focused purely on HFpEF. We have now reflected that in the text.

We have edited the inclusion criteria to emphasise our inclusive approach.

22) 1If you considered any type of PA, then it would seem reasonable to include studies that focussed on self-care, of which PA is an important component? Later you state that you included studies that described perceptions of HF patients living with their illness, if this is the case then a large body of studies would seem to be missing from the review? (see table added as attachment)

Our aim was to identify studies with content from people with heart failure describing their participating in physical activity. We defined physical activity.

We knew that some studies would be directly focused on physical activity and that there would also some studies in which only part of the study would describe such data. At abstract screening stage we were very inclusive in our selection including self-care and living with heart failure studies. Indeed, as can be seen in our search strategy we included self-care terms.

At full paper stage for most papers, it was very clear if there was relevant content or not. There were a few borderline cases which we discussed in the screening team (LD, SD, ALH) and if needed with other members of the team. As a general rule we looked for a section or theme on physical activity within a paper to allow inclusion. We did not include studies which had relevant but isolated individual quotes.

We have edited the inclusion criteria section to reflect the above, being clear on what was not stated explicitly in the Prospero protocol.

We have added in out definition of physical activity which was included in the Prospero protocol.

Cross-refencing the list provided. Our searches found the 3 most recent reviews in our searches although not the Welstand 2009 integrative review – possibly due to poorer indexing of this older, less rigorous review. Thus, we screened the content of Austin 2021, Jeon 2010 and Falk 2013 for relevant studies as part of our systematic review. In total our initial search found 35 systematic reviews and our update an additional four systematic reviews , not all relevant but many, were in part relevant and so their content was screened. **We did not formally record this process as these papers were excluded at the first screening, but we have now added these numbers to our PRISMA flowchart and edited the text in the methods to clarify this.**

We are therefore confident in our searches and our inclusion of studies.

Interestingly, our initial unscreened search results in Rayaan included 20% of the original studies in the list provided from these past reviews but equally only 7% of our included studies are in these four previous reviews in the provided table. This reflects two things- the volume of qualitative studies focusing on people with heart failure and the importance of having a defined scope on any review of this literature. The scope of our review was very clear around its focus on experiences of participation in physical activity by people with heart failure. Added to that, we found our 2024 update added little in terms of new findings and had no impact on our categories or synthesised findings. We are confident in our synthesised findings and conclusion of our final systematic review and meta-aggregation.

23) The synthesised findings expressed as statements lack deep description, likely due to the volume of work that the authors have presented within this report, therefore it is difficult to really understand the substance/depth/breadth behind these statements.

As described in our general statement we have slimmed down other aspects of this paper to allow us to have more word count to focus on the synthesised

findings. This has allowed us to describe these synthesised findings in more depth.

Some particular comments are presented below:

24) Results 1: Physical symptoms. It is not clear whether this synthetises construct is limited to heart failure symptoms; it would seem that it is not? This should be expanded to clarify is possible, most patients with HF will have multi-morbidity (quote 1) which may explain a lot of symptomology?

We agree with this statement. We have revised this first synthesised finding with greater depth which has allowed us to talk about the impact of comorbidities.

25) Results 2: Emotional Factors. What symptoms are people fearful of exacerbating, breathlessness, pain, MSK issues? All quotes related to fear, it is difficult to verify the 'motivation', could this be added as an alternative? The quote presented in theme 3 extrinsic factors about 'being overwhelmed' hints that emotional factors could be more than just fear of worsening symptoms, it seems like psychological factors such as self-efficacy and resilience might also be at play?

We have revised this synthesised finding and added more detail. This has allowed us to explain emotional factors better, giving a clearer message We have also cross-referenced the synthesised findings in our text. In other words, acknowledging that some factors and phenomena are relevant to more than one synthesised finding.

26) Result 3: Extrinsic factors. Were other factors like the beliefs of others not important here? Quite a lot of literature on activity in heart failure states that the beliefs of others can modify engagement both positively and negative (i.e. Tierney and Smith) Is the quote on asthma more about their co-existing conditions rather than the weather i.e. concurrent asthma modifies their response to the weather, rather than the weather per se - perhaps this belongs in the physical symptoms section?

Again, by acknowledging that the content of the final synthesised findings are relevant to more than one synthesised finding we hope that we have acknowledged the complexity of the experiences of people with heart failure.

27) Result 4. note typo 'reenforces' should be reinforces. Is this also about safety and not just knowledge?

This has been corrected.

We have added detail to clarify the findings on both safety and knowledge.

28) Result 5. As above, does this not overlap with extrinsic factors?

As before, we have acknowledged the crossover with the more detailed descriptions we have given.

29) Result 6. Are the experiences of pain, fear and breathlessness reported earlier not also negative responses to activity? There are also quotes to suggest that being worse off financially is a potential negative consequence.

As above.

We have added text to reflect financial impact and other factors. Due to word count we have had to limit representational quotes for some factors.

Discussion:

30) Overall the discussion is quite short and with the exception of the comparison to the Cochrane cardiac rehab review, it does not compare the findings to other studies, should this be expanded?

Agree – we have expanded on this. In general, research on physical activity and rehabilitation with HF has focused on intervention, efficacy and functional outcomes but we acknowledge that there is a body of qualitative research including some reviews. We have cited and contextualised the most relevant and recent reviews and have also restated our rationale for the review, meta-aggregation and logic model which is based on the gap in the evidence, and in real life a gap in support for PWHF.

31) The review does not include cardiac rehabilitation studies, which is open to people with HF. Cardiac rehab has a long history of offering remote and digital physical activity via structures rehabilitation pathways, if these studies were included, would it change conclusions relating to technological/digital abilities?

We did include CVR studies if they included qualitative data as per our inclusion criteria. Of our 14 clinical studies at least half could be described as rehab or structured exercise programs. Many of these interventions involved technology in various ways e.g. telehealth (remote provision), exergames, fitness trackers.

The aim of our review and MA was to determine what PWHF were experiencing and perceived around PA. There was not a focus on digital/tech support. Looking back at the original findings – this topic does not come up at all in the purely qualitative studies but that is not surprising when many of them are quite broad-brush or very specific e.g. sex life, fatigue . In the intervention studies there was of course acceptability and feasibility questions posed to the participants around digital/tech intervention e.g. exergaming, yoga on DVD , telerehab and fit bit. Overall, they were acceptable and pWHF for example liked not having to leave their own home, prompts were motivating but these are also countered with 'gets boring after a while', internet not working/difficult to set up etc The aim and process of our meta-aggregation does not really do justice to this topic area and so no clear message came through our synthesis.

We have added text to explain this in the discussion and to give a more rounded view point on digital/tech support but like all EDI issues this problem is multilayered- there is what happens in the real world and what happens in a clinical trial and what criteria is laid down in terms of inclusion criteria for trials. Digital facilitation and digital exclusion is a very important topic area for care of PWHF and goes beyond support for PA – we have edited our discussion to reflect that as well.

32) The conclusions about embedding support in the community does not link obviously (at least not to me), from the findings of the review (synthesised statements). Whilst some activity is better than none, it is not clear that 'PA in everyday life', is sufficient to improve symptoms and quality of life in HF? Current evidence suggests that HIIT and continuous training are more optimal approaches for improving cardiorespiratory fitness, are these approaches compatible with 'PA in everyday life'? Future interventions need to be cognisant of the many barriers as highlighted, but they also need to acknowledge the clinical evidence base which highlights these approaches.

Agree and we have edited and added to the detail of the motivation of our review and meta-aggregation which is in brief the point that access to rehab is limited in the UK for many PWHF esp. the older mainly community-cared for

patients. Despite the known benefits of rehab and the tailoring of interventions (if eligible) uptake and completion is still modest. This is coupled with the fact despite the existence of rehab – the number 2 JLA PSP question suggests that PWHF in the community are not confident in their PA and feel that the HCPs are not confident in their support or advice. We believe our revised discussion contextualises our ongoing research better. – Thank you for you really useful critique.

As comment 14) Overall this is a very large piece of work and the authors should be congratulated for the robust approaches they have adopted. However, I feel that presenting everything within the one report limits the readers' ability to appreciate the breadth and depth of the synthesised statements, as they are minimally described in order to allow for the integration of the other components of the review within in word limit of the journal.

Please see our general statement at the beginning of our responses. We believe the paper is very improved by your comments.

Response to Reviewer: 3 Dr. Christine Brown Wilson, Queen's University Belfast

Thank you for the opportunity to review this paper that outlines the development of a logic model from a systematic review and met-aggregation of qualitative data alongside a study within a review process for consideration of EDI factors. The involvement of a PPI group in this process is a strength of this work and well documented for others to follow.

As comment 8) The only suggestion I would make would be to explain the process of meta aggregation as opposed to a general synthesis of the data. Overall the process leading to the findings is well documented with clear tables. The data presented in the findings underpin each theme with a clear link to the logic model, which has also been presented for a non academic audience. The discussion clearly outlines the issues that have been raised with clear recommendations for future studies. I hope you find these comments helpful.

As for response to comment 8) we have added Figure 2 with the detail of the meta-aggregation process.
